# Distinct pseudokinase domain conformations underlie divergent activation mechanisms among vertebrate MLKL orthologues

**DOI:** 10.1038/s41467-020-16823-3

**Published:** 2020-06-19

**Authors:** Katherine A. Davies, Cheree Fitzgibbon, Samuel N. Young, Sarah E. Garnish, Wayland Yeung, Diane Coursier, Richard W. Birkinshaw, Jarrod J. Sandow, Wil I. L. Lehmann, Lung-Yu Liang, Isabelle S. Lucet, James D. Chalmers, Wayne M. Patrick, Natarajan Kannan, Emma J. Petrie, Peter E. Czabotar, James M. Murphy

**Affiliations:** 1 Walter and Eliza Hall Institute of Medical Research, 1G Royal Parade, Parkville, VIC 3052 Australia; 2 Department of Medical Biology, University of Melbourne, Parkville, VIC 3052 Australia; 3 Institute of Bioinformatics, University of Georgia, Athens, GA 30602 USA; 4 Department of Biochemistry, University of Otago, Dunedin, 9054 New Zealand; 5 School of Biological Sciences, Victoria University of Wellington, Wellington, 6140 New Zealand

**Keywords:** Kinases, X-ray crystallography, Necroptosis

## Abstract

The MLKL pseudokinase is the terminal effector in the necroptosis cell death pathway. Phosphorylation by its upstream regulator, RIPK3, triggers MLKL’s conversion from a dormant cytoplasmic protein into oligomers that translocate to, and permeabilize, the plasma membrane to kill cells. The precise mechanisms underlying these processes are incompletely understood, and were proposed to differ between mouse and human cells. Here, we examine the divergence of activation mechanisms among nine vertebrate MLKL orthologues, revealing remarkable specificity of mouse and human RIPK3 for MLKL orthologues. Pig MLKL can restore necroptotic signaling in human cells; while horse and pig, but not rat, MLKL can reconstitute the mouse pathway. This selectivity can be rationalized from the distinct conformations observed in the crystal structures of horse and rat MLKL pseudokinase domains. These studies identify important differences in necroptotic signaling between species, and suggest that, more broadly, divergent regulatory mechanisms may exist among orthologous pseudoenzymes.

## Abstract (web summary)

The necroptotic cell death pathway involves signaling through pseudokinases. Here the authors define the structural determinants of species specificity in necroptosis signaling mediated by the essential necroptotic effector pseudokinase, Mixed Lineage Kinase Domain-Like (MLKL).

## Introduction

The necroptosis cell death pathway is thought to have originated as an altruistic innate immunity mechanism involved in host defense against pathogens^1–5^. The recent implication of pathway dysregulation in the pathology of many human diseases, including inflammatory diseases^6–8^, ischaemic reperfusion injuries^9,10^ and inflammatory bowel disease^11^, has led to increased interest in targeting necroptosis therapeutically. The necroptosis pathway can be triggered by a number of stimuli, including death ligands, such as tumour necrosis factor (TNF), and pathogen-derived stimuli, such as lipopolysaccharides^12^. In scenarios where the activities of the E3 ligase family, the inhibitors of apoptosis proteins (IAPs), and the apoptotic proteolytic enzyme, Caspase-8, have been compromised, the protein kinases, RIPK1 and RIPK3, are incorporated into a large molecular weight complex termed the “necrosome”^13–15^. Within the necrosome, RIPK3 is believed to be activated by autophosphorylation^16,17^ before promoting the executioner function of the terminal effector in the pathway, the Mixed Lineage Kinase domain-Like (MLKL) pseudokinase^18–20^.

MLKL contains an N-terminal four-helix bundle (4HB) domain, which enables MLKL’s membrane translocation^21^ and is responsible for the plasma membrane permeabilization that characterizes necroptotic cell death^18,22–25^. The 4HB domain executioner function is regulated by the C-terminal pseudokinase domain^25^, which serves as a receiver for upstream signals, such as activation loop phosphorylation by RIPK3^18,19,26,27^. Pseudokinase domains are catalytically defective owing to the absence of key catalytic motifs known to mediate phosphoryl transfer in conventional protein kinases^28–32^. Instead these domains serve functions as signal integrators and/or protein interaction modules^31,32^. RIPK3-mediated MLKL phosphorylation has been proposed to provoke a conformational change in the pseudokinase domain that is transmitted to the 4HB domain via a two-helix linker termed the brace helices^18,25,33^. By this mechanism, MLKL undergoes a conversion from the dormant basal, cytoplasmic monomer into higher-order entities that translocate to, and mediate, plasma membrane compromise^25,33–35^.

Here, we sought to shed light on the mechanisms governing necroptotic signaling across the vertebrate phylogenetic tree. Recently revealed differences between necroptosis signaling in mouse and human cells^12,15,33,34,36^ indicate that the precise mechanism of MLKL activation by RIPK3, and how they might vary across vertebrates, remains incompletely understood. Mouse MLKL activation relies on transient engagement of RIPK3 to facilitate phosphorylation of the pseudokinase domain^25–27^, while it appears that stable recruitment of human MLKL by necrosomal RIPK3 is an additional crucial step in human MLKL activation^5,34^. We sought to establish the molecular determinants that govern RIPK3 recognition of MLKL by examining which MLKL orthologues can communicate with the necroptosis machinery in mouse and human cell lines. Although the capacity of pseudokinase orthologues to complement signaling pathways in other species has not been widely examined, here our studies reveal remarkable selectivity among orthologues, with pig MLKL able to be activated by human RIPK3 and horse MLKL by mouse RIPK3. Unexpectedly, despite its high sequence identity to mouse MLKL, rat MLKL was not able to reconstitute necroptotic signaling in mouse cells. The molecular basis for RIPK3 selectivity could be rationalized from knowledge obtained from the crystal structures of the rat and horse MLKL pseudokinase domains. The inability of rat MLKL to complement the mouse necroptosis pathway could be attributed to the respective active kinase-like and inactive kinase-like conformations of the rat and mouse MLKL pseudokinase domains. The capacity of horse MLKL to communicate with mouse RIPK3 is enabled by an additional helix N-terminal to the αC helix in horse MLKL, which spatially occupies the position of the mouse activation loop helix in the mouse MLKL:mouse RIPK3 structure. Additionally, the activation loop of horse MLKL adopts an inactive-like conformation that nestles within the pseudoactive site. These findings raise the possibility that activation loop phosphorylation may promote activation loop mobility, and destabilise inactive MLKL conformations, to facilitate molecular switch interconversion and MLKL dissociation from RIPK3.

## Results

### Mouse and human RIPK3 selectively activate MLKL orthologues

**Fig. 1:**
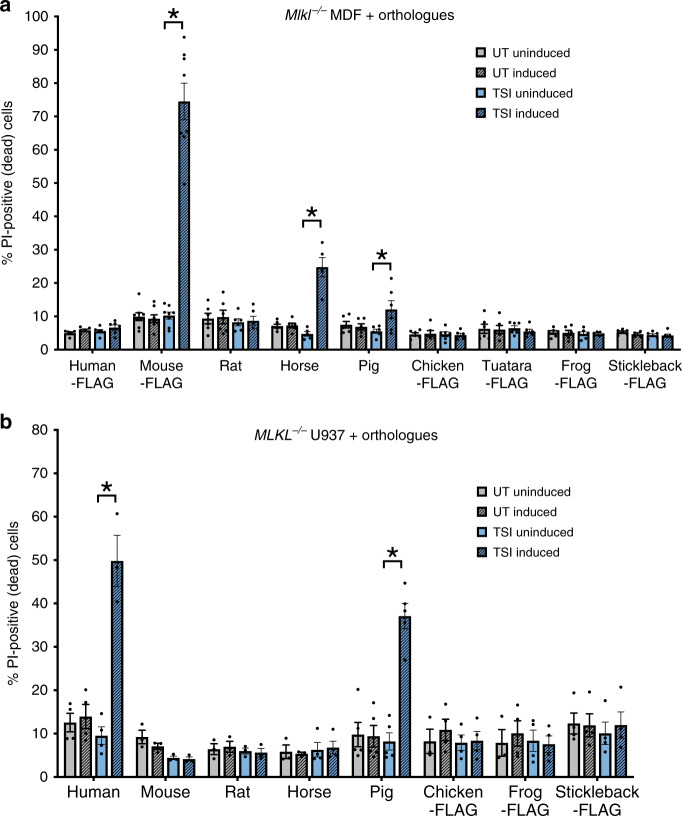
Few MLKL orthologues reconstitute necroptotic signaling in mouse and human cells. Genes encoding human, mouse, rat, horse, pig, chicken, tuatara, frog and stickleback MLKL were stably introduced into *Mlkl*^*−/−*^ mouse dermal fibroblast (MDF) (**a**) and *MLKL*^*−/−*^ human U937 (**b**) cells and expressed upon doxycycline treatment (induced). Cells were either untreated (UT) or treated with a necroptotic stimulus (TNF, Smc mimetic, IDN-6556; TSI) to examine the capacity of each orthologue to reconstitute necroptotic signaling. Cell death was measured by propidium iodide (PI) uptake by flow cytometry. Data shown are mean ± SEM of independent experiments on one U937 cell line (*n* = 3 for mouse, rat, horse and chicken MLKL; *n* = 4 for human and frog MLKL; *n* = 5 for pig MLKL) or two biological replicate MDF lines (*n* = 6, except for *n* = 8 for mMLKL-FLAG). * represents statistical significance of *p* < 0.05 using a paired, two-tailed *t*-test: **a** mouse-FLAG *p* = 0.000015, horse *p* = 0.0017, pig *p* = 0.0290; and **b** human *p* = 0.0104, pig *p* = 0.0003. Source data are provided in a Source Data file. An example of the flow cytometry gating strategy used throughout this study is shown in Supplementary Fig. 2. Expression of introduced genes was verified by western blot (Supplementary Fig. 3).

To examine the compatibility of orthologous MLKL proteins with the necroptosis machinery in mouse and human cells, we introduced genes encoding human (*Homo sapiens*), mouse (*Mus musculus*), rat (*Rattus norvegicus*), horse (*Equus caballus*), pig (*Sus scrofa*), chicken (*Gallus gallus*), stickleback (*Gasterosteus aculeatus*), frog (*Xenopus tropicalis*) and tuatara (*Sphenodon punctatus*) MLKL into *Mlkl*^*−/−*^ mouse dermal fibroblasts (MDFs) and *MLKL*^*−/−*^ human U937 cells. These orthologues were chosen to maximize our sampling of phylogenetic diversity among vertebrate MLKL sequences in nature, ranging in sequence identity from 35%–85% to mouse and 36%–65% to human MLKL (Supplementary Fig. 1, Table 1). Orthologue MLKL constructs were stably introduced into these cell lines via a puromycin-selectable lentiviral vector^18^, from which MLKL expression could be induced using doxycycline (dox). Cells were stimulated with TNF, Smac mimetic and the Caspase inhibitor, IDN-6556/emricasan (TSI), to initiate necroptosis, as described before^34,36^, in the presence or absence of dox-induced orthologous MLKL gene expression. Cell death was measured by flow cytometry using propidium iodide (PI)-uptake (exemplified in Supplementary Fig. 2). Owing to sequence divergence, existing MLKL antibodies did not recognize all orthologue sequences. To enable verification of expression by western blot, chicken, stickleback, frog and tuatara MLKL were C-terminally FLAG-tagged (Supplementary Fig. 3a, b), because N-terminal tags are known to compromise the killing function of mouse and human MLKL^23,25^. Mouse MLKL bearing a C-terminal FLAG tag reconstituted necroptotic signaling in *Mlkl*^*−/−*^ MDF cells (Fig. 1a), supporting the notion that C-terminal tagging does not compromise MLKL function. Among orthologues tested, only mouse and horse, and to a lesser extent pig, MLKL orthologues could reconstitute signaling in *Mlkl*^*−/−*^ mouse fibroblasts, with death only observed when orthologue expression was induced and the necroptosis stimulus, TSI, applied. Notably, horse MLKL killed cells less potently than the mouse counterpart, and pig MLKL was only able to induce low levels of cell death (12%) upon treatment with the necroptosis stimulus, TSI. Remarkably, given the high sequence identity to mouse MLKL (86% identical; 96% similar) and the high similarity of rat and mouse RIPK3 sequences (Tables 1 and 2), rat MLKL did not kill mouse cells (Fig. 1a). Expression of MLKL orthologues in human *MLKL*^*−/−*^ U937 cells revealed only human and pig MLKL could reconstitute necroptotic signaling in human cells (Fig. 1b). This was also surprising, because human MLKL shows greater sequence similarity to the horse protein than to pig MLKL (Table 1). These data also confirm that mouse MLKL could not substitute for human MLKL in the human necroptosis pathway, as previously inferred from immunoprecipitation studies in which human MLKL did not interact with mouse RIPK3 (ref. ^37^). We note that while we could verify expression of the MLKL orthologues by immunoblot (Supplementary Fig. 2a–d), it is not possible to estimate the relative abundance of each protein. Sequence divergence precludes the use of the same antibody to detect expression of each orthologue owing to epitope differences, and even where antibodies cross-react, such as for the anti-MLKL 3H1 clone, the epitopes differ between the mouse, rat, human and horse sequences. Additionally, it remains to be established if there is a threshold level of MLKL expression required for the execution of necroptotic cell death, and whether yet-to-be-identified co-effectors may differ between species and influence the kinetics of cell death upon reconstitution with orthologues. Together, our studies underscore the remarkable selectivity of RIPK3 orthologues for their cognate MLKL effectors, consistent with the idea that RIPK3–MLKL cassettes have co-evolved in response to selective pressures, such as those exerted by pathogens^5^.

**Table 1:** Pairwise amino acid sequence identity and similarity of MLKL orthologues over full-length sequence and component domains.

Species	Mouse identity (similarity) %			Human identity (similarity) %		
	Full length	4HB + brace	Pseudokinase	Full length	4HB + brace	Pseudokinase
Mouse	–	–	–	61.8 (84.0)	51.7 (82.6)	69.3 (86.6)
Rat	85.8 (96.3)	82.7 (97.1)	87.9 (96.2)	62.2 (84.2)	53.9 (80.3)	68.1 (87.9)
Pig	60.2 (83.2)	52.4 (77.5)	66.1 (87.3)	63.0 (88.6)	57.8 (87.2)	68.3 (91.8)
Horse	63.0 (83.3)	56.1 (78.1)	67.6 (87.1)	65.3 (88.3)	57.9 (87.4)	70.2 (89.0)
Chicken	39.4 (66.6)	31.7 (66.5)	50.8 (74.8)	40.2 (70.4)	32.4 (69.0)	50.5 (76.0)
Tuatara	39.8 (70.4)	29.1 (69.8)	49.1 (76.2)	41.0 (73.6)	30.5 (70.2)	52.9 (79.7)
Frog	36.0 (71.1)	23.5 (68.7)	45.7 (74.3)	35.9 (65.8)	23.8 (64.5)	45.2 (70.3)
Stickleback	35.1 (65.9)	28.1 (63.7)	41.8 (71.1)	35.8 (67.8)	24.2 (62.9)	44.4 (71.2)

Alignments performed with lalign (https://www.ebi.ac.uk/Tools/psa/lalign/)^70^.

**Table 2:** Pairwise amino acid sequence identity and similarity of full length or kinase domains of RIPK3 orthologues.

Species	Mouse identity (similarity) %		Human identity (similarity) %	
	Full length	Kinase domain	Full length	Kinase domain
Mouse	–	–	60.0 (76.1)	70.0 (85.7)
Rat	76.4 (88.0)	82.1 (91.6)	60.4 (76.7)	70.8 (84.3)
Pig	58.6 (77.1)	69.3 (85.3)	62.7 (79.6)	71.2 (85.8)
Horse	60.9 (78.0)	71.7 (87.3)	64.5 (80.1)	74.2 (87.4)
Chicken	–	–	–	–
Tuatara	40.2 (67.5)	42.0 (67.9)	41.2 (65.7)	43.8 (68.0)
Frog	33.8 (59.5)	38.4 (64.9)	33.8 (62.1)	38.2 (64.8)
Stickleback	23.2 (53.6)	n/a	23.2 (53.6)	n/a

Alignments performed with lalign (https://www.ebi.ac.uk/Tools/psa/lalign/)^70^.

**Fig. 2:**
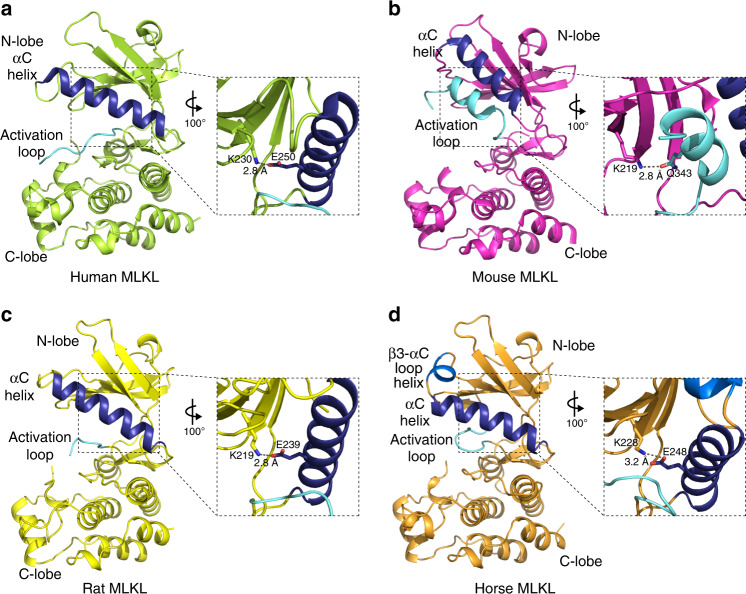
Rat and horse MLKL pseudokinase domain structures diverge from mouse MLKL. **a** The structure of the human MLKL pseudokinase domain (PDB, 4MWI^39^) shows a conventional active kinase-like conformation. The K230–E250 salt bridge (equivalent of the K72-E91 PKA interaction) between the β3 ATP-binding lysine and the Glu in the αC helix (shown in dark blue throughout) is shown in the zoomed inset. **b** The mouse MLKL pseudokinase domain (from PDB 4BTF^18^) shows a more open conformation owing to the activation loop adopting an unusual helical conformation (pale blue), which displaces the αC helix (dark blue). An unconventional interaction between the β3 K219 and the activation loop Q343 results (zoomed inset). **c** The rat MLKL pseudokinase domain adopts an active protein kinase-like conformation, resembling that of the human structure. The β3 K219 forms a conventional salt bridge with the αC helix E239 (zoomed inset), rather than a hydrogen bond with the activation loop Gln observed in the mouse structure. **d** The horse MLKL pseudokinase domain shows a similar active protein kinase-like conformation, with the K228:E248 salt bridge shown in the zoomed inset. The horse structure exhibits previously unobserved features, including burial of the activation loop in the pseudoactive site and an additional helix in the β3-αC loop (displayed in more detail in Fig. 4).

### Rat and horse MLKL exhibit conformational heterogeneity

It was of interest to understand why horse, but not rat, MLKL could reconstitute necroptotic signaling in *Mlkl*^*−/−*^ MDFs, despite the high sequence identity of mouse and rat MLKL sequences. To obtain molecular insights into this selectivity, we crystallized the pseudokinase domains of rat (residues 179–464) and horse MLKL (residues 188–475), following their expression and purification from insect cells, and solved their crystal structures at 2.2 and 2.7 Å resolution, respectively (Fig. 2, Supplementary Table 1). Both structures exhibit typical features of the pseudokinase/kinase domain fold: a smaller, N-terminal lobe comprising principally β-strands and a larger C-lobe composed predominantly of α-helices. The disposition of the αC helix in each structure was typical of that of a conventional (catalytically active) protein kinase. The conserved Glu of the αC helix in either structure forms a salt bridge with the β3-strand Lys of the VAIK motif, which contributes to assembly of an intact regulatory (R)-spine^38^.

We next compared the rat and horse MLKL pseudokinase domain structures to those of mouse and human MLKL previously reported^18,39^ (Fig. 2a–d). The mouse MLKL pseudokinase domain contains a distinguishing activation loop helix that abuts the αC helix (Fig. 2b) and displaces it from participating in interactions present within the active sites of conventional protein kinases^18^. In particular, in mouse MLKL, Q343 in the activation loop helix forms a hydrogen bond to K219 of the β3-strand VAIK motif, precluding engagement of the VAIK motif Lys (K219) in a conventional ion pair with the αC helix Glu (E239). This contrasts the human MLKL pseudokinase domain, which contains the conventional VAIK-Lys:αC helix Glu interaction (K230:E250) and exhibits an intact R-spine to resemble an active protein kinase structure^39^ (Fig. 2a). Based upon overall structural features, such as the αC helix position and the VAIK-Lys:αC helix Glu ion pair, the rat and horse MLKL pseudokinase domain structures reported herein more closely resemble the human MLKL pseudokinase, with respective RMSD of 0.962 and 0.712 Å across Cα atoms. Considering the high sequence similarity between rat and mouse MLKL, this is surprising, and the structural divergence of rat and mouse MLKL pseudokinase domains may explain why rat MLKL cannot reconstitute the necroptosis signaling pathway in mouse cells lacking *Mlkl*.

While grossly topologically similar to rat and human MLKL pseudokinase domain, the horse MLKL pseudokinase domain structure revealed unexpected features (Fig. 2d). In the N-lobe, we observed an unconventional helix in the β3-strand-αC helix loop that is positioned adjacent to the αC helix between S233 and I238. Additionally, although typically unstructured in protein kinase and pseudokinase structures, a substantial portion of the activation loop (residues L351-I358) was well resolved in the horse MLKL pseudokinase domain structure (Supplementary Fig. 4). This loop folded back from a conventional position adjacent to the αC helix towards the hinge and was buried within the “pseudoactive”/ATP-binding pocket. Crucially, two key residues, T356 and S357, are buried in this cleft (Fig. 2d, cyan), and these residues correspond to the residues phosphorylated by RIPK3 in human MLKL (T357 and S358), whose phosphorylation are widely considered hallmarks of MLKL activation.

### Horse MLKL β3-αC loop helix facilitates mouse RIPK3 binding

**Fig. 3:**
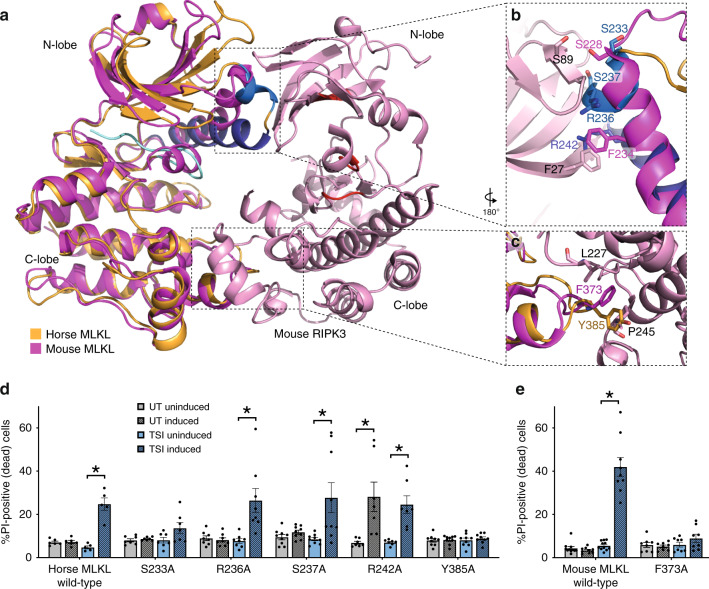
Horse MLKL β3-αC loop and C-lobe residues facilitate mouse RIPK3 engagement. **a** The horse MLKL pseudokinase domain structure superimposed on the reported mouse MLKL pseudokinase:mouse RIPK3 kinase domain structure (PDB 4M69 (ref. ^40^)). A site in each of the N- and C-lobes of horse MLKL pseudokinase domain were identified as possible interfaces between horse MLKL and mouse RIPK3 (**b**, **c**). **d** Wild type or alanine substitutions of putative interface residues in horse MLKL were expressed in *Mlkl*^*−/−*^ MDF cells and their capacity to induce death in the absence of stimulation (untreated, UT) or upon addition of a necroptotic stimulus (TSI) following doxycycline induction of protein expression was quantified by PI uptake. **e** Wild type or F373A mouse MLKL were expressed in *Mlkl*^*−/−*^ MDF cells and their capacity to induce death evaluated as in panel **d**. Data in **d**, **e** are shown are mean ± SEM of ≥3 independent experiments for two biological replicate MDF lines (*n* = 6 for wild-type horse MLKL; *n* = 7 for S233A, R241A; *n* = 8 for R235A, F373A; and *n* = 9 for S237A, Y384A and mouse MLKL wild type). * represents statistical significance of *p* < 0.05 using a paired, two-tailed *t*-test: **d** horse MLKL wild type *p* = 0.0017, R236A *p* = 0.0162, S237A *p* = 0.0249, R242A UT uninduced vs UT induced *p* = 0.0236, R242A TSI uninduced vs TSI induced *p* = 0.0041; **e** wild-type mouse MLKL *p* = 0.0002. Source data are provided in a Source Data file.

To deduce how horse MLKL could communicate with mouse RIPK3 to induce its activation in *Mlkl*^*−/−*^ mouse fibroblasts, we superimposed the horse MLKL pseudokinase domain structure upon the mouse MLKL pseudokinase domain within the previously described mouse MLKL:RIPK3 kinase domain crystal structure^40^ (Fig. 3a). We observed that the β3-αC loop helix, only observed in horse MLKL to date, spatially occupied the position of the mouse MLKL αC helix, leading us to hypothesize a role for this helix in mediating RIPK3 recognition. In particular, S233 of horse MLKL is structurally proximal to S228 of mouse MLKL, which forms a hydrogen bond with S89 of mouse RIPK3 in the MLKL:RIPK3 co-crystal structure^40^ (Fig. 3b). We also postulated that R242 of horse MLKL may recapitulate the π–π stacking interaction of F27 (mouse RIPK3) and F234 (mouse MLKL) with a cation–π stacking interaction, and therefore also contribute to the N-lobe RIPK3 binding interaction. The intervening residues in horse MLKL, R236 and S237, were also oriented to face the RIPK3 N-lobe in the structural overlay (Fig. 3b). Therefore, we generated Ala substitution mutants of the predicted RIPK3 interactors, S233, R236, S237 and R242 (Fig. 3b), within full-length horse MLKL and examined whether their expression in mouse *Mlkl*^*−/−*^ MDF cells could induce cell death in the presence or absence of necroptotic stimuli (Supplementary Fig. 3, Fig. 3d). Each mutant could reconstitute the necroptosis pathway in *Mlkl*^*−/−*^ mouse fibroblasts except S233A horse MLKL, while R242A horse MLKL exhibited constitutive activity in the absence of a necroptotic stimulus. These data support a role for the novel β3-αC loop helix structurally positioning specific residues of the horse MLKL N-lobe to facilitate the mouse RIPK3 interaction. We speculate that this conformation, which is a distinguishing feature of horse MLKL, precludes interaction with human RIPK3 and contributes to species specificity.

Within the C-lobe of MLKL, we recently implicated the hydrophobic residue, F384, of human MLKL in the engagement of human RIPK3 for necroptotic signaling in human U937 cells^5^. Here, we observed that Y385 of horse MLKL and F373 of mouse MLKL are positioned within the αEF-αF loop in analogous positions to facilitate mouse RIPK3 engagement (Fig. 3c). To test this idea, we introduced Ala substitutions of Y385 in full-length horse MLKL and F373 in full-length mouse MLKL and examined their capacity to reconstitute the necroptosis pathway when expressed in *Mlkl*^*−/−*^ MDF cells (Fig. 3d, e). These substitutions completely abrogated necroptotic signaling, supporting a conserved function for this aromatic residue in contributing to the RIPK3 binding interface. Collectively, our mutational analysis of horse MLKL indicates that the acquisition of an atypical N-lobe helix that mimics the unusual mouse MLKL αC helix position enables selective activation of horse MLKL by mouse RIPK3. In addition, mutation of horse MLKL Y385 and mouse MLKL F373 supports the previously proposed idea^5^ that this aromatic residue, conserved in many MLKL orthologues, but not stickleback and frog MLKL (Supplementary Fig. 1), is essential for the C-lobe RIPK3 interaction.

### Function of MLKL phosphorylation differs amongst orthologues

**Fig. 4:**
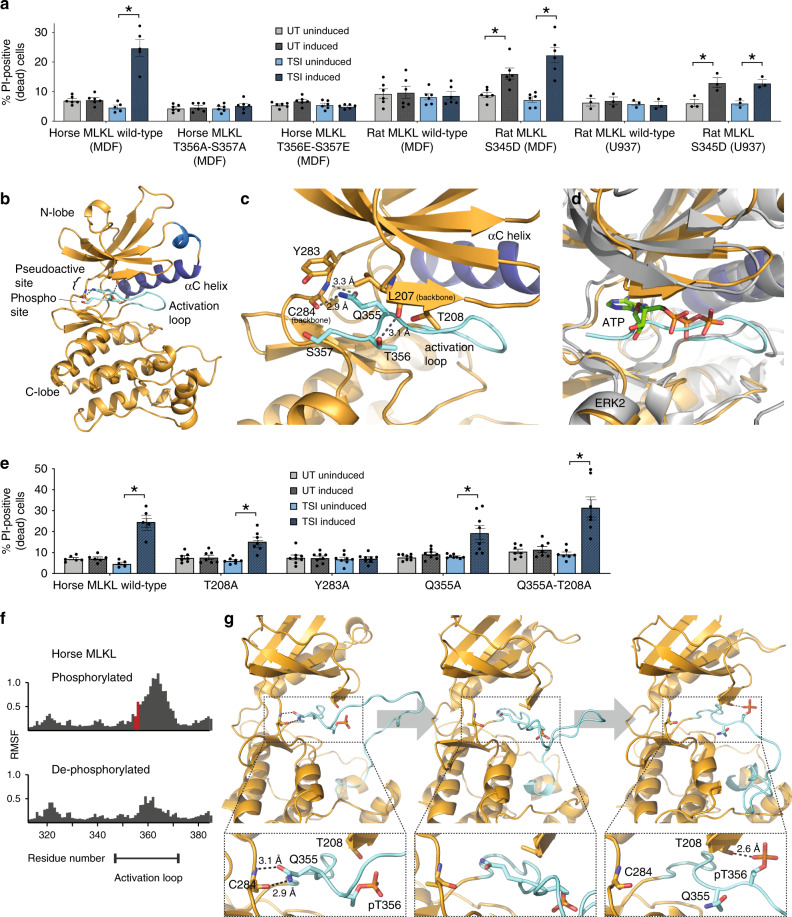
Horse MLKL activation loop phosphorylation induces conformational flexibility. **a** The activity of phosphomimetic mutations, S345D in rat MLKL, or T356E-S357E in horse MLKL, or alanine substitution (T356A-S357A) in horse MLKL, was tested in *MLKL*^*−/−*^ U937 and/or *Mlkl*^*−/−*^ MDF cells upon doxycycline induction of expression, in the presence (TNF, Smac mimetic, IDN-6556; TSI) and absence (untreated; UT) of necroptotic stimuli. These data are plotted alongside wild-type controls from Fig. 1. **b**, **c** The activation loop of the horse MLKL pseudokinase domain is buried in the pseudoactive site in a position occupied by ATP in conventional protein kinases, such as ERK (PDB 4GT3) (**d**). **e** Wild-type horse MLKL or alanine substitution mutants of activation loop and adjacent pseudoactive site residues were stably introduced into *Mlkl*^*−/−*^ MDF cells and the capacity to kill cells in the presence (TSI) or absence (UT) of necroptotic stimuli in the presence (induced) or absence (uninduced) of doxycycline-induced exogene expression quantified by PI uptake using flow cytometry. Data in **a** and **e** are shown as mean ± SEM of ≥3 independent experiments for each of two biological replicate MDF lines (*n* = 6 for all in **a**, *n* = 7 for T208A and Q355A-T208A, *n* = 8 for Y282A and Q355A) or one U937 line (*n* = 3). * represents statistical significance of *p* < 0.05 using a paired, two-tailed *t*-test: **a** wild-type horse MLKL *p* = 0.0017, rat MLKL S345D in MDFs UT uninduced vs UT induced *p* = 0.0089, rat MLKL S345D in MDFs TSI uninduced vs TSI induced *p* = 0.0035, rat MLKL S345D in U937s UT uninduced vs UT induced *p* = 0.0108 and rat MLKL S345D in U937s TSI uninduced vs TSI induced *p* = 0.00114. For **e** T208A *p* = 0.0013, Q355A *p* = 0.0099 and Q355A-T208A *p* = 0.0056. **f** A comparison of molecular dynamics simulations on horse MLKL reveals increased activation loop flexibility in the phosphorylated MLKL model. The *x*-axis shows residue numbers and the *y*-axis shows root mean square fluctuation (RMSF) across the simulation. The phosphorylated residues, pT356 and pS357, are shown in red. **g** A series of snapshots of phosphorylated horse MLKL show the phosphorylated activation loop moving out of the pseudoactive site. Zoomed insets show hydrogen bonds at various stages of the transition.

RIPK3-mediated phosphorylation of the MLKL pseudokinase domain activation loop is a recognized hallmark of necroptosis pathway activation^18,19,27,41^. However, the precise function it serves in MLKL activation remains unclear. In the case of mouse MLKL, activation loop phosphorylation on S345 is thought to be sufficient to convert MLKL into a killer protein^18,26^. By contrast, phosphomimetic or phospho-ablating mutations of the activation loop residues, T357 and S358, prevented human MLKL’s participation in necroptosis signaling, presumably by prohibiting RIPK3-mediated activation^34^. Here, we sought to test whether rat and horse MLKL behave like mouse MLKL, where phosphomimic mutants can trigger constitutive cell death, or like human MLKL, where mutation of phosphosites blocks killing. We introduced the S345D mutation into rat MLKL to mimic substitution in its mouse MLKL counterpart (Supplementary Fig. 3e). When inducibly expressed in *Mlkl*^*−/−*^ MDF or *MLKL*^*−/−*^ U937 cells, cell death was significantly elevated ~2-fold above background levels of death (Fig. 4a). These data support the idea that a phosphomimetic substitution in the rat MLKL pseudokinase domain can trigger interconversion to a pro-necroptotic state, albeit less effectively than the counterpart mutation within mouse MLKL. In horse MLKL, we introduced Ala or Glu substitutions of T356 and S357, the horse counterparts of the RIPK3 substrates in human MLKL, T357 and S358^19^, and found that these mutations completely abrogated the capacity of these constructs to participate in necroptotic signaling in mouse cells (Fig. 4a). The lack of activity by the Ala substitution mutant suggests that the activity of horse MLKL in mouse cells is reliant on RIPK3-mediated phosphorylation of those residues. The phosphomimetic data suggest that, unexpectedly, the horse MLKL T356E-S357E mutant behaves more similarly to the human MLKL phosphomimetic mutant, which is inactive, than the constitutively active mouse counterpart.

In our horse MLKL pseudokinase domain structure, we observed nestling of the activation loop within the pseudoactive/ATP-binding site (Fig. 4b–d). To our knowledge, such an activation loop orientation in the ATP-binding cleft has not been reported in earlier pseudokinase or kinase domain structures. More broadly, such a conformation raises the prospect that MLKL might sequester its activation loop within the ATP-binding site, and phosphorylation by RIPK3 might trigger the conformational change that underlies transition of MLKL from dormant monomer to a pro-necroptotic oligomer. Accordingly, we mutated residues in the activation loop and proximal residues in the “pseudoactive” site of horse MLKL to explore their role in necroptotic signaling. Crucially, unlike mouse MLKL^18^, mutation of the activation loop residue equivalent to Q343 in mouse MLKL, horse MLKL Q355, which mediates H-bonding between the activation loop and the hinge region in the horse MLKL structure, did not impact necroptotic signaling (Fig. 4e). The rotamer of T356 could not be assigned with certainty in our structure, and appeared to form a hydrogen bond with either the backbone carbonyl group of L207 or the hydroxyl group of the T208 side chain in different chains of the asymmetric unit. Mutation of T208 to Ala did not perturb the function of horse MLKL in mouse cells, which may indicate that T356 hydrogen bonds with the backbone of L207, or that the N-lobe:Q355 and T356 activation loop hydrogen bonds that stabilize the activation loop in this conformation are not essential for horse MLKL function (Fig. 4e). We further explored the molecular basis for these observations by subjecting the pseudokinase domain of horse MLKL to molecular dynamics simulations.

Unbiased simulations of the phosphorylated and dephosphorylated horse MLKL were performed starting from the crystallized conformation in which the activation loop is buried in the pseudoactive site, with the unresolved portion of the activation loop (residues 358–366) modelled using RosettaRemodel. The buried conformation can be characterized by hydrogen bonds between the C284 peptide backbone and the activation loop Q355 carboxamide (Fig. 4c, g, left). While this interaction remains stable in dephosphorylated MLKL, it is unstable in the pT356/pS357 MLKL simulations, as indicated by the high root mean square fluctuations of the activation loop residues in the phosphorylated form in comparison to the dephosphorylated form (Fig. 4f). Furthermore, analysis of simulation snapshots depicts the phosphorylated activation loop moving out of the pseudoactive site to an extended conformation in which the Q355–C284 interaction is broken (Fig. 4g). These data suggest that the addition of phosphate groups destabilizes the buried activation loop conformation and support the concept that, among some MLKL orthologues, activation loop phosphorylation may induce increased mobility to promote RIPK3 dissociation following its phosphorylation of MLKL. Further exploration of this idea in other orthologues, such as human MLKL, using molecular dynamics simulations is difficult with currently available structures, because the activation loop residues subject to phosphorylation are unresolved. Despite the experimental challenges, it remains of immense interest whether phosphorylation-induced activation loop mobility is a generalized feature of MLKL activation by RIPK3.

## Discussion

Pseudokinases, and pseudoenzymes more generally, commonly serve functions as regulators of their catalytically active enzyme partners^30–32,42^. Cognate pseudoenzymes are thought to have arisen from gene duplications of active enzymes, where the duplicate is relieved of selective pressures to enable evolution of new, non-catalytic functions, often within the same pathway^43^. It is typical of pseudokinases, like the TYK2 JH2 domain, KSR2, STRADα and HER3/ERBB3 to bind and regulate the activities of their cognate active kinases: the TYK2 JH1 domain, MEK1, LKB1 and EGFR, respectively^44–47^. However, the case of MLKL and its active partner kinase, RIPK3, is unusual among known pseudoenzyme:enzyme pairs. Rather than the pseudokinase MLKL regulating the activity of RIPK3, it is RIPK3 that controls MLKL activation via engagement and phosphorylation of the MLKL pseudokinase domain^18,19,26,27,34^. MLKL is also an unusual pseudokinase owing to its rapid evolution^48,49^. Sequence divergence among orthologues is thought to have arisen from selective pressures, such as those applied by pathogens to evade necroptotic death as a host defense measure^1,4,5,50,51^. Here, we have provided a dramatic illustration of how the pairwise interaction between MLKL’s pseudokinase domain and the RIPK3 protein kinase domain has co-evolved between species. We examined the capacity of human and mouse RIPK3 to activate human, mouse, rat, horse, pig, frog, tuatara, chicken and stickleback MLKL orthologues, and the underlying structural basis for the observed selectivity. Until now, the molecular basis for selectivity among different species was poorly understood, with few determinants of species specificity deduced. In previous studies, human MLKL was unable to reconstitute necroptotic signaling in *Mlkl*^*−/−*^ mouse cells^36^, but this could be overcome by swapping the pseudokinase domain of human MLKL for the mouse counterpart^33^. Within RIPK3, the αG helix in the C-lobe, which includes the phosphorylated mouse T231/S232 and human S227 residues, is known to be crucial for recognition of their cognate MLKL orthologues^5,37^. However, with only the mouse RIPK3 kinase domain structure reported to date^40^, interpretation of the selectivity of RIPK3 for different MLKL orthologues remains challenging considering similar extents of sequence diversity among orthologues as observed for MLKL (Table 2).

Our data revealed a remarkable interspecies divergence of RIPK3:MLKL cognate pairs, such that the mouse and human RIPK3 substrate range for activation of MLKL orthologues is highly restricted. Our data indicate that mouse, horse and, to a lesser extent, pig MLKL could be activated by mouse RIPK3, while only human and pig MLKL could be activated by human RIPK3. Activity in both cell lines was unique to pig MLKL, which showed a strong preference for human RIPK3 activation. While our capacity to interpret the selectivity of human RIPK3 for human and pig MLKL is limited by the unavailability of the human RIPK3 kinase domain and pig MLKL structures, we could rationalize the selectivity of mouse RIPK3 for mouse and horse MLKL, in preference to rat MLKL, based on their structures. It remains to be addressed whether the pig MLKL β3-αC loop adopts a helical structure like that observed in our horse MLKL pseudokinase domain structure. The two MLKL orthologues share similar sequences within this loop—S^233^QARS in horse, S^237^QASD in pig—raising the prospect that both might form helices. It is notable that this helix is very dynamic in horse MLKL molecular dynamics simulations. Consequently, establishing the presence of a comparable helix in the pig MLKL, and whether it might contribute to interaction with human RIPK3 in U937 cells, awaits an experimental structure. Collectively, our mutational data support three crucial interfaces between the MLKL pseudokinase domain and the RIPK3 kinase domain that mediate a face-to-face interaction: one each between the N-lobes, the C-lobes, and the activation loops. In the MLKL pseudokinase domain C-lobe, we identified a highly conserved Phe/Tyr residue in the αEF-αF loop, based on the projection of F373 of mouse MLKL into a pocket in the C-lobe of mouse RIPK3 kinase domain within the complex structure. Previously, we observed the human MLKL F386 and poxviral MLKL F202 counterparts of mouse MLKL F373 were crucial to human RIPK3 interaction^5^. Here, we observed that F373 of mouse MLKL and Y385 of horse MLKL were similarly crucial to mouse RIPK3 engagement. Our findings support a conserved role for this αEF-αF loop aromatic residue in RIPK3 interaction, and thus do not suggest a function for this residue in dictating the species specificity of RIPK3:MLKL interactions. This aromatic residue is widely conserved among MLKL orthologues, including chicken and tuatara, but present as Asn in most birds, frogs, lizards and fishes. Similar modes of engagement have been described between other pseudokinase:kinase cognate pairs (reviewed in ref. ^52^), consistent with the notion that pseudokinase C-lobe projection into cognate kinase domain C-lobe cavities have convergently evolved as common interaction and regulatory determinants.

While it is clear that the MLKL pseudokinase domain αEF-αF loop aromatic residue is an anchor for RIPK3 recognition, its widespread conservation indicates that selectivity for RIPK3 engagement must be at least partially dictated by other sites within MLKL. Previous mutational analyses of mouse MLKL implicated the αC helix residue, S228, as a contributor to RIPK3 regulation^27^. Here, we identified the counterpart in horse MLKL, S233, as an important residue for mouse RIPK3 recognition. This residue is spatially conserved in horse MLKL owing to the occurrence of a short helix in the β3-αC loop that is not observed in other MLKL pseudokinase domain structures. Such structural conservation is absent from the rat MLKL pseudokinase domain structure, which likely accounts for its inability to substitute for mouse MLKL in the mouse necroptosis pathway despite ~88% sequence identity between rat and mouse MLKL pseudokinase domains. We also identified horse MLKL R242 as a potential determinant of mouse RIPK3 binding. This residue occupies a similar position to the mouse MLKL residue, F234, which was previously proposed to participate in the mouse RIPK3 binding interface by inspection of the complex structure^40^. Unexpectedly, however, Ala substitution of horse MLKL R242 led to its autoactivation, where expression alone induced cell death in the absence of a necroptotic stimulus. There are several possible explanations for the constitutive cell death induced by R242A horse MLKL. One possibility is that the R242A mutation enables constitutive activation via mouse RIPK3-mediated phosphorylation of horse MLKL, although currently this is not possible to test experimentally owing to the unavailability of suitable antibodies for phospho-horse MLKL. Another possibility is that the R242A substitution prompts a conformational change in the horse MLKL pseudokinase domain that unleashes the MLKL’s killer 4HB domain, or facilitates exposure of MLKL to necroptosis co-effectors^53–55^, to enable cell death to ensue. In contrast to mouse MLKL, where mutations in the “pseudoactive” site can trigger constitutive cell death, the horse MLKL pseudokinase domain adopts a conventional active-like kinase domain fold similar to human MLKL. Consequently, we expect horse MLKL to behave similarly to human MLKL, where pseudoactive site mutations compromise, rather than promote, killing activity. Collectively, these data implicate the position of the αC helix and adjacent structural elements in the MLKL pseudokinase domain as important factors in dictating the propensity for RIPK3 interaction.

The third site we have implicated in RIPK3 interaction is the activation loop of the MLKL pseudokinase domain. Our data indicate that the activation loop can serve two clear, but not mutually exclusive, functions in regulating MLKL killing activity. On the one hand, introduction of acidic residues to mimic RIPK3 phosphorylation within the activation loop of the MLKL pseudokinase domain triggered stimulus-independent activation of mouse MLKL^18,26,27,53^. Similar substitutions abrogated killing by human MLKL even in the presence of necroptotic stimuli^34^. This suggests a second function of activation loop phosphorylation: to serve as a cue for disengagement from necrosomal RIPK3. In keeping with the latter idea, viral MLKL proteins inhibit cellular RIPK3 via a constitutive association, which cannot be relieved by RIPK3 phosphorylation owing to their contracted activation loops lacking appropriate RIPK3 substrate residues^5^. Here, we mutated sites in the activation loops of horse and rat MLKL orthologous to the RIPK3 substrates in human and mouse MLKL, respectively. Introduction of the S345D mutation into rat MLKL modestly triggered its killing function in both mouse and human MLKL-deficient cells, suggesting that rat MLKL activation follows principles similar to mouse MLKL activation. Whether S345D rat MLKL would induce more profound death in rat cells, owing to a reliance on endogenous species-specific co-effectors, remains of outstanding interest. Curiously, mutation of the horse activation loop did not promote constitutive killing, and abrogated its capacity to reconstitute necroptotic signaling in *Mlkl*^*−/−*^ mouse cells, indicating activation loop phosphorylation serves a function similar to that attributed to human MLKL. Consistent with this notion, molecular dynamics analyses suggest destabilization of the buried activation loop conformation following phosphorylation could perturb interaction with RIPK3 and may promote MLKL dissociation from the necrosome.

Therefore, while MLKL’s pseudokinase domain structure, mode of RIPK3 interaction, the function of MLKL activation loop phosphorylation and the stoichiometries of MLKL assemblies^33,34^ are heterogeneous between species, there are unifying principles underlying MLKL activation. Principally, RIPK3-mediated phosphorylation of the MLKL pseudokinase domain activation loop, the assembly into higher-order species, and the translocation of MLKL oligomers to the plasma membrane, are hallmarks of necroptosis. Our data support the idea that the MLKL pseudokinase domain can act variously as an integrator of phosphorylation events, interconvert as a molecular switch, and mediate protein–protein interactions, albeit with differences evident in the underlying mechanisms between species. We anticipate that future studies will reveal more examples of pseudoenzymes that display functional heterogeneity between orthologues to allow them to fulfil signal transduction requirements via variant mechanisms, as we have observed for MLKL.

## Methods

### Genes, antibodies and reagents

DNA sequences were synthesized to encode MLKL from the following species: horse (accession XP_005608486; Bioneer, South Korea), rat (accession XP_003753159; ATUM, CA), pig (accession XP_003481839; ATUM, CA), chicken (accession XP_015134716; Bioneer, South Korea), tuatara^56^ (Ensembl accession ENSSPUT00000023207.1; Bioneer, South Korea), stickleback (Ensembl accession ENSGACT00000011307.1; GeneArt, Germany), frog (accession XP_002931711; Bioneer, South Korea), in addition to previously described human and mouse MLKL genes (DNA2.0, CA)^18,34^. All sequences are available upon request. DNA sequences were introduced into the doxycycline-inducible, puromycin-selectable vector, pF TRE3G PGK puro^18,25,36^. Pig, stickleback, frog, tuatara, human and mouse MLKL genes were cloned into a derivative vector containing an in-frame C-terminal FLAG tag. Mutations were introduced into the wild-type templates using oligonucleotide-directed overlap PCR. All insert sequences were verified by Sanger sequencing (Micromon DNA Sequencing Facility, VIC, Australia).

Primary antibodies used in this study were rat anti-MLKL (clone 3H1, produced in-house;^18^ 1:1000 dilution; available as MABC604; EMD Millipore, Billerica, MA, USA) for all MLKL blots except for pig MLKL, which was detected with rat anti-MLKL clone 5C4 (produced in-house, 1:25 dilution of unpurified hybridoma supernatant); mouse anti-Actin (A-1987; Sigma-Aldrich, St Louis, MO, USA; 1:3000); rabbit anti-GAPDH (cat#2118; Cell Signaling Technology, Danvers, MA; 1:3000); and rat anti-FLAG (clone 9H1, produced in-house; 1:1000). Secondary goat anti-mouse (cat#1010-05), goat anti-rabbit (cat#4030-05) and goat anti-rat Ig-HRP conjugates (cat#3010-05) were supplied by Southern Biotech and used at 1:5000 dilution. Recombinant hTNF-Fc, produced in-house, and the Smac mimetic, Compound A, were used as reported earlier^57,58^. The pan-caspase inhibitor, IDN-6556/emricasan, was provided by Tetralogic Pharmaceuticals.

### Protein expression and purification

Horse (residues 188–475) and rat (residues 179–464) MLKL pseudokinase domains were cloned into pFastBac Htb and expressed in *Sf*21 insect cells using the Bac-to-Bac (ThermoFisher) system, using procedures established for other MLKL pseudokinase domain orthologues^18,29,39^. Briefly, expressed proteins were purified by Ni-NTA chromatography (Roche) and the His_6_ tag cleaved by TEV protease treatment to leave a GAMGS overhang. Following dialysis and further Ni-chromatography, the proteins were eluted from Superdex-200 gel filtration chromatography in 0.2 M NaCl, 20 mM HEPES pH 7.5, 5% glycerol and concentrated by centrifugal ultrafiltration to 5 mg/mL.

### Protein crystallization and structure determination

Rat and horse MLKL pseudokinase domain were subjected to sparse matrix screening in sitting drops containing 150 nL protein and 150 nL of reservoir solution at 20 °C (C3 Facility; CSIRO, Parkville, VIC). Rat MLKL was crystallized in 20 mM MnCl_2_, 25% v/v PEG400, 0.1 M sodium MES pH 6.5 and crystals flash frozen in liquid N_2_ using PEG400 in the mother liquor as the cryoprotectant. Horse MLKL was crystallized in 2 M ammonium sulfate, 0.1 M sodium HEPES pH 7.5 and crystals flash frozen following transfer to 2 M sodium malonate pH 7. Diffraction data were collection at the MX2 beamline of the Australian Synchrotron using an Eiger detector, at 100 K, with a wavelength of 0.9537 Å. Data reduction, integration and scaling was performed using XDS^59^. The human MLKL pseudokinase domain coordinates (PDB 4MWI^39^) were used as the search model in Phaser-MR^60^ in Phenix^61^ for both structures. Refinement was carried out in Phenix.refine iteratively with model building in Coot^62^. The final model Ramachandran statistics were 96.6% favoured, 3.4% allowed and 0% outliers for the rat MLKL pseudokinase structure, and 96.2% favoured, 3.8% allowed and 0% outliers for the horse MLKL pseudokinase structure. Final statistics for refined structures are presented in Supplementary Table 1.

### Cell line generation and expression constructs

pF TRE3G puro pgk vectors encoding MLKL variants were co-transfected into HEK293T cells with pVSVg and pCMV δR8.2 helper plasmids to generate lentiviral particles^18,25^. These lentiviruses were used to introduce doxycycline-inducible MLKL constructs into biologically independent MDF lines derived from three *Mlkl*^*−/−*^ mice and immortalized using SV40 large T antigen^18,25^. MDF cells were cultured in DMEM (Life Technologies) supplemented with 10% (v/v) foetal calf serum (FCS), penicillin (100 U/mL), streptomycin (100 μg/mL) and, during and after selection for vector integration, puromycin (5 μg/mL). *MLKL*^*−/−*^ U937 cells^34^ were cultured in human tonicity RPMI medium (prepared in-house) supplemented with 8–10% v/v FCS and puromycin (5 μg/mL) for lines stably transduced with MLKL expression constructs. Protein expression was validated by western blot using established methods^5,18,34^.

### Cell death assays

Cell death assays were performed using established methods^18,25,34^. Briefly, MDFs were seeded at 5 × 10^4^ cells/well into 24-well plates, and allowed to adhere overnight. Cells were treated with 10 ng/mL doxycycline, and after 3 h either left untreated, or treated with TNF (100 ng/mL), Smac mimetic (Compound A; 500 nM) and IDN-6556 (5 μM) (TSI) or Q-VD-OPh (10 μM) (TSQ). After 24 h, cells were harvested and treated with propidium iodide (PI; 100 ng/mL), and PI-positive cells counted by BD FACSCalibur flow cytometry. U937 cells were assayed equivalently, but for the addition of doxycycline (10 ng/mL) immediately after plating.

### Molecular dynamics simulations

The solved crystal structure of horse MLKL was used as the starting conformation for molecular dynamics simulations. Disordered residues in the activation loop (358–366) were modelled using RosettaRemodel^63^. Post-translational modifications were introduced using PyTMs^64^. Unbiased all-atom molecular dynamics simulations were performed using GROMACS 2016.4 (ref. ^65^). Structures were parameterized using the CHARMM36 (ref. ^66^) force field and solvated with the TIP3P water model. Random solvents molecules were replaced with sodium or chloride ions to neutralize the charge of the system. The system was contained in a dodecahedron at least 1 nm larger than the protein from all sides with periodic boundary conditions. Long-range interactions were calculated with particle mesh Ewald. Neighbour lists were maintained using the Verlet cutoff scheme^67^. The system underwent steepest descent minimization until the maximum force was <100 kJ/mol. Canonical ensemble^68^ was used to heat the system from 0 to 310 K in 100 ps. Isothermal–isobaric ensemble^69^ (1 bar, 310 K) was applied for 100 ps. Positional restraints were applied during equilibration. Production runs used 2 fs time steps. Phosphorylated and dephosphorylated MLKL were simulated for 2882 and 1739 ns, respectively.

### Reporting summary

Further information on research design is available in the Nature Research Reporting Summary linked to this article.

## Supplementary information

Supplementary Information

Peer Review

Reporting Summary

## Data Availability

Atomic coordinates for the rat and horse MLKL pseudokinase domains have been deposited in the Protein Data Bank with the accession numbers PDB 6VBZ and PDB 6VC0, respectively. Other data are available from the corresponding authors upon request. The data underlying Fig. 1, Fig. 3d and e, Fig. 4a and e and Supplementary Fig. 3 are provided as a Source Data file. Source data are provided with this paper.

## Source data

Source Data
