# Supplementary material for: Distinct pseudokinase domain conformations underlie divergent activation mechanisms among vertebrate MLKL orthologues
Source: Nat Commun. 2020 Jun 19;11:3060. doi: 10.1038/s41467-020-16823-3 (PMC7305131; doi:10.1038/s41467-020-16823-3)
Supplement: Supplementary file 1 — Supplementary Information [file 41467_2020_16823_MOESM1_ESM.pdf]

SUPPLEMENTARY INFORMATION

Distinct pseudokinase domain conformations underlie divergent activation mechanisms among vertebrate MLKL orthologs

Davies et al.

Supplementary Table 1. **X-ray crystallography data collection and refinement statistics**

	Rat MLKL (179-464)	Horse MLKL (188-475)
Data collection		
Space group	P 6_1 *2 2*	C *2*
Cell dimensions		
a, *b*, *c* (Å)	190.0, 190.0, 77.79	155.5, 124.8, 81.05
α, β, γ (°)	90, 90, 120	90, 112.7, 90
Resolution (Å)	47.5 – 2.19 (2.27 – 2.19)	40.1 – 2.75 (2.84 – 2.75)
R-merge	0.1024 (1.24)	0.1914 (1.548)
$I / \sigma I$	24.83 (3.04)	8.79 (0.99)
Completeness (%)	100 (98)	99 (90)
Redundancy	20.2 (20.3)	6.9 (6.2)
Refinement		
Reflections used in refinement	42667 (4080)	36830 (3346)
R-work	0.1852 (0.2348)	0.1834 (0.3138)
R-free	0.2161 (0.2913)	0.2379 (0.3601)
No. non-hydrogen atoms	4300	6645
Protein	4112	6585
Ligand	6	12
Water	182	48
Average B-factor	51.33	59.53
Protein	51.21	59.44
Ligand	91.83	65.59
Water	52.50	45.13
R.M.S. deviations		
Bond lengths (Å)	0.01	0.01
Bond angles (º)	1.02	1.38

Statistics for the highest-resolution shell are shown in parentheses.

SUPPLEMENTARY FIGURES

Supplementary Figure 1. Amino acid sequence alignments for MLKL orthologs examined in this work.

Sequence alignment of orthologs with Linnaean names on the left and common names on the right. Residues conserved with identity (dark green) and high similarity (light green) are highlighted in the alignment. Regions equivalent to catalytic motifs on canonical kinases are coloured red, and residues of the αC helix (dark blue) and activation loop (light blue) are highlighted. The position of the horse β3-αC loop helix is indicated in bright blue, and the residues equivalent to RIPK3 binding residue (F373 in mouse) are highlighted in yellow. The domain architecture of mouse MLKL (PDB:4BTF) is illustrated above the alignment: lines are loops, cylinders are helices and arrows β-sheets. Underlined, bolded residues are RIPK3 substrates in mouse and human MLKL. Residue numbering corresponds to the mouse MLKL sequence.

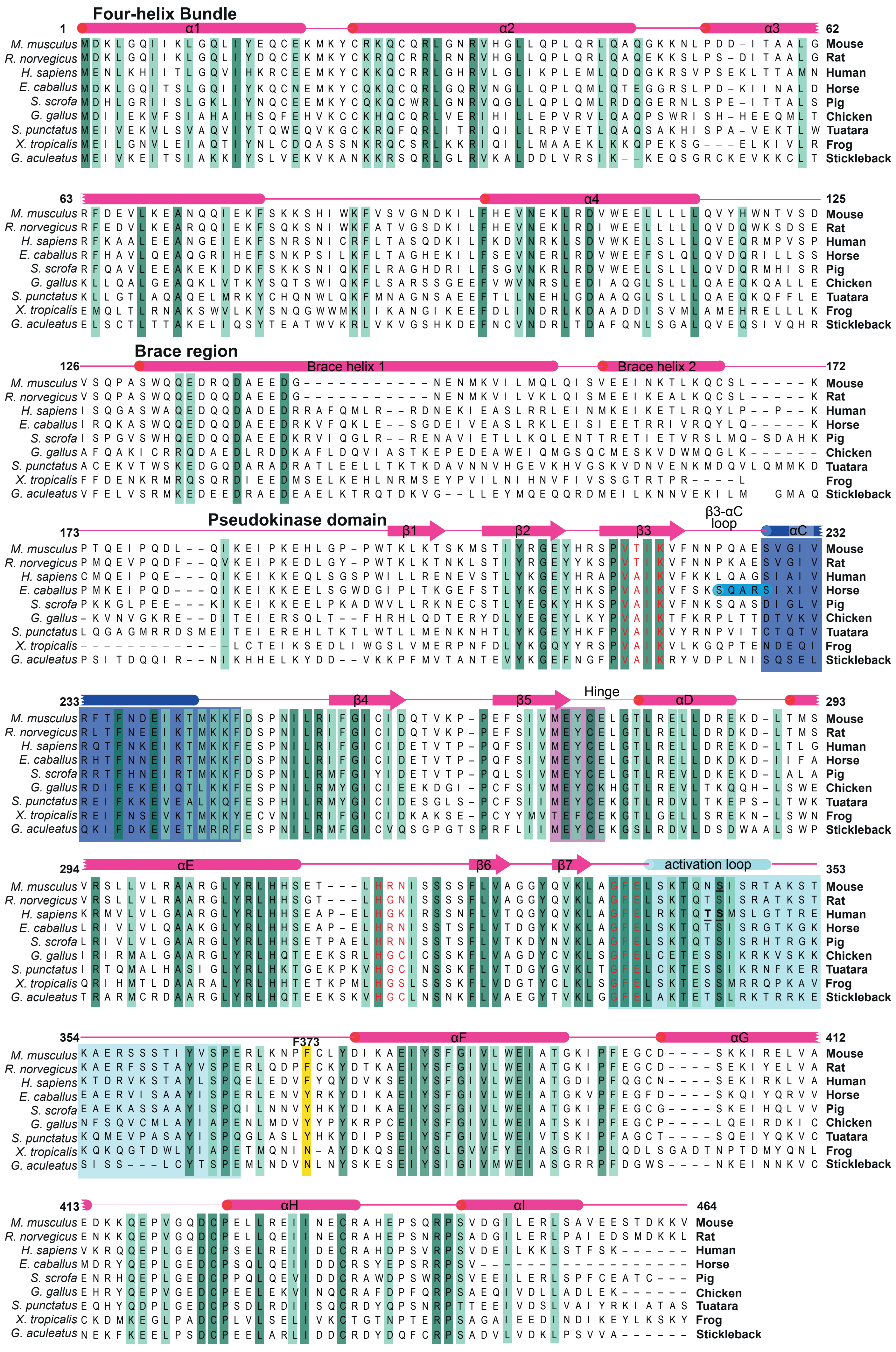
Four-helix Bundle
α1
α2
α3
α4
Brace region
Brace helix 1
Brace helix 2
Pseudokinase domain
β1
β2
β3
β3-αC loop
αC
β4
β5
Hinge
αD
αE
β6
β7
activation loop
F373
αF
αG
αH
αI
M. musculus
R. norvegicus
H. sapiens
E. caballus
S. scrofa
G. gallus
S. punctatus
X. tropicalis
G. aculeatus
Mouse
Rat
Human
Horse
Pig
Chicken
Tuatara
Frog
Stickleback

Supplementary Figure 2. An example of the flow cytometry gating strategy used throughout this study.

Intact cells were gated as shown in (a). Subsequently, viability was measured based on propidium iodide (PI)-uptake (b), where PI uptake identified dead cells. This strategy has is as previously reported[1].

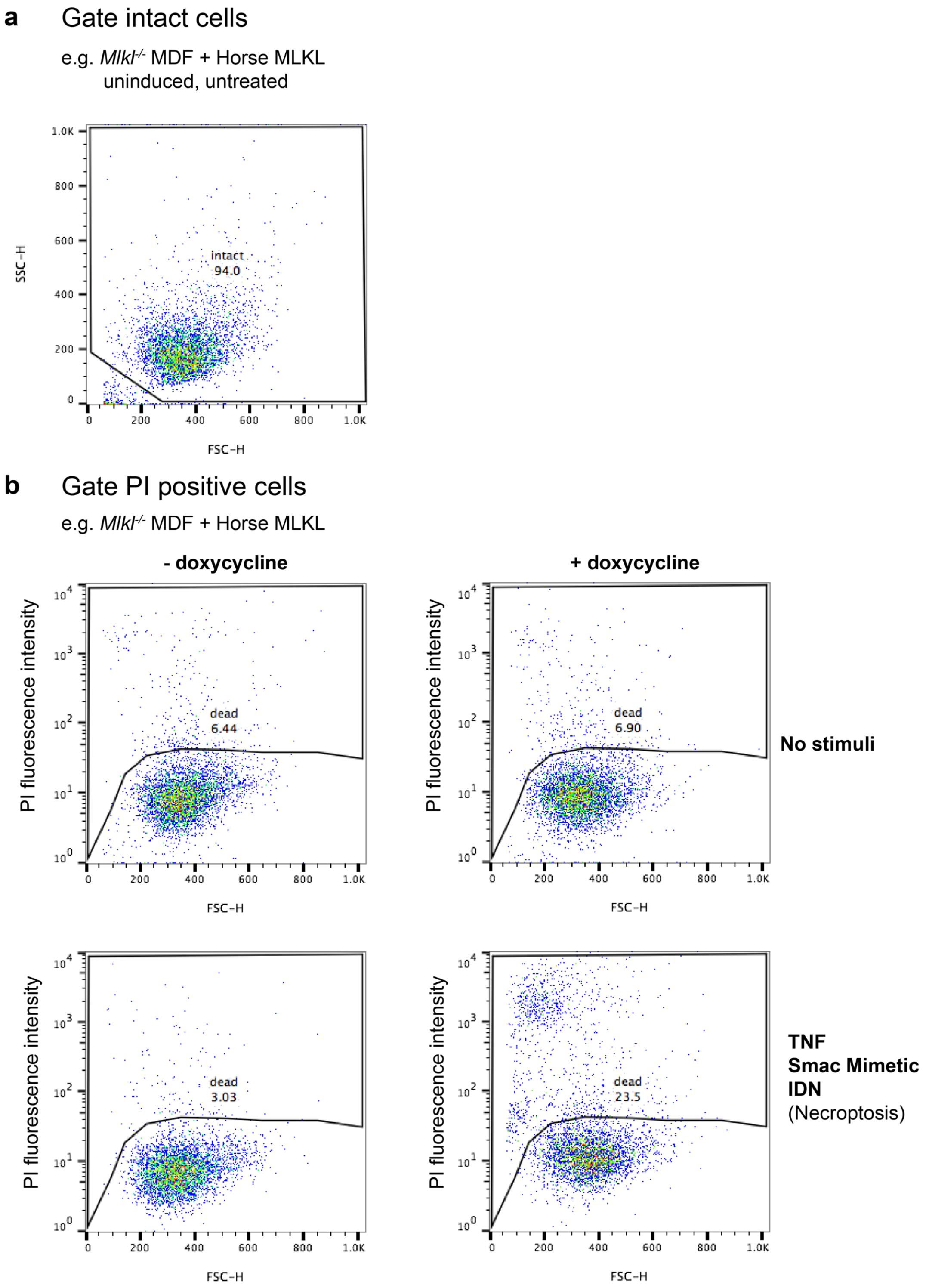

Supplementary Figure 3. Western blot validation of MLKL ortholog and mutant expression in *Mlkl*$^{-/-}$ mouse dermal fibroblasts (MDF) and *MLKL*$^{-/-}$ U937 human cells.

(**a**) Doxycycline-induced expression of mouse, human, horse, rat, tuatara, chicken, stickleback, pig and frog MLKL in *Mlkl*$^{-/-}$ MDF cells was validated by western blot using the anti-MLKL mAb (clone 3H1), via a C-terminally fused FLAG tag or using another anti-MLKL mAb (clone 5C4) for pig MLKL. Exogenes were expressed in MDF lines derived from biologically-independent *Mlkl*$^{-/-}$ mice (denoted 391, 392 and 393). GAPDH blots were used as loading controls. (**b**) Expression of orthologs in *MLKL*$^{-/-}$ human U937 cells was monitored by western blot as for MDF cells in (a). Note tuatara MLKL expression was not detectable in U937 cells. (**c, d**) Expression of horse and mouse MLKL mutants in *Mlkl*$^{-/-}$ MDF cells was monitored by western blot as described in (a). (**e**) Rat S345D mutant MLKL expression in *Mlkl*$^{-/-}$ MDF and *MLKL*$^{-/-}$ U937 cells was validated with the pan-MLKL mAb (3H1 clone) by western blot. The positions of molecular weight markers are shown on the left in each panel. The shown western blots are representative of 2 independent repeats. Uncropped blots are provided in a Source Data file.

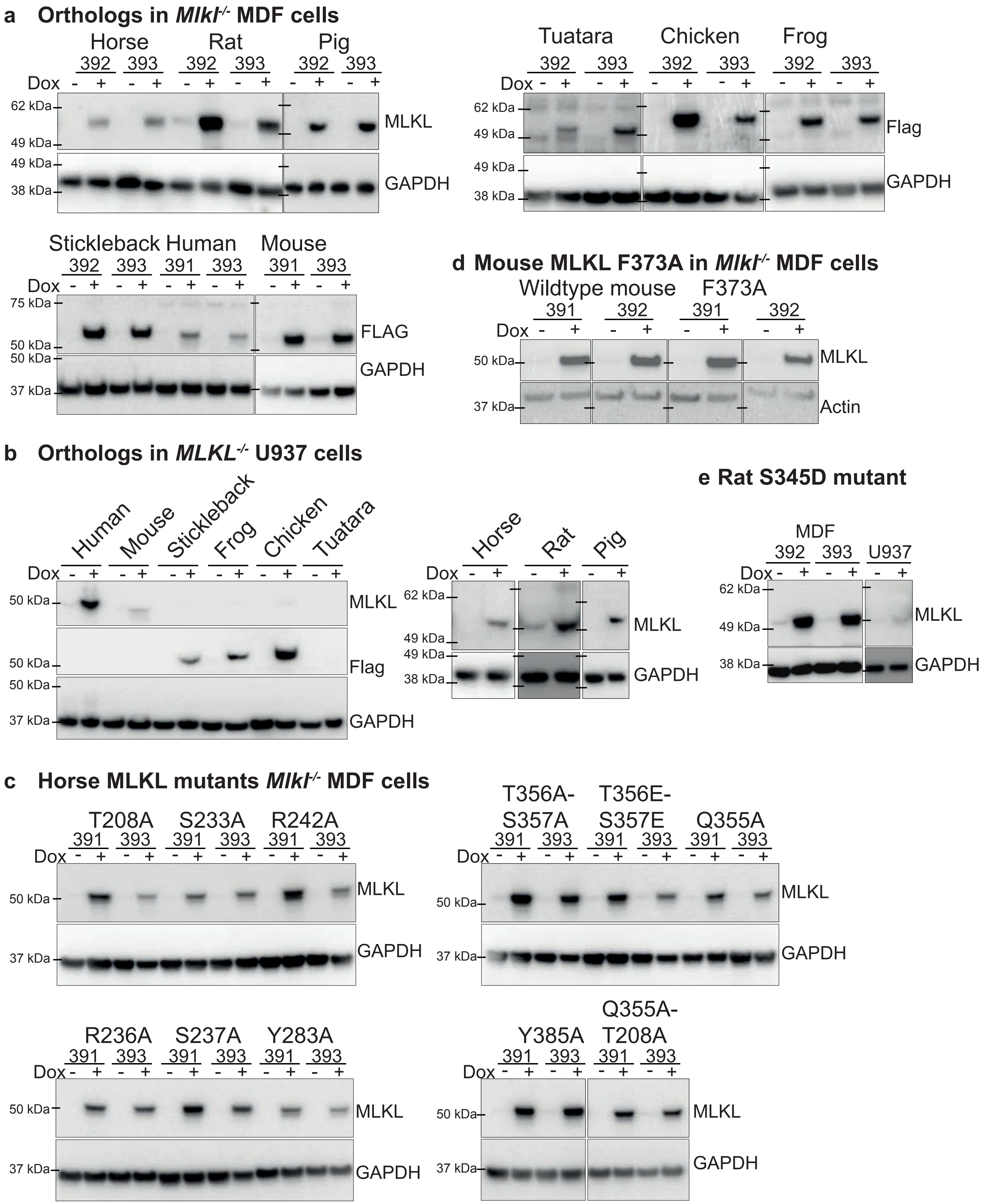
a Orthologs in Mlkl-/- MDF cells
Horse
Rat
Pig
392
393
Dox
MLKL
GAPDH
62 kDa
49 kDa
38 kDa
Tuatara
Chicken
Frog
Flag
Stickleback
Human
Mouse
391
FLAG
75 kDa
50 kDa
37 kDa
d Mouse MLKL F373A in Mlkl-/- MDF cells
Wildtype mouse
F373A
Actin
b Orthologs in MLKL-/- U937 cells
e Rat S345D mutant
MDF
U937
c Horse MLKL mutants Mlkl-/- MDF cells
T208A
S233A
R242A
T356A-S357A
T356E-S357E
Q355A
R236A
S237A
Y283A
Y385A
Q355A-T208A

Supplementary Figure 4. Electron density maps for the novel activation loop conformation of horse MLKL.

Electron density maps for the activation loop and surrounding regions of the horse MLKL pseudokinase structure for all three chains of the aysmmetric unit. The blue mesh shows the σ weighted 2Fo-Fc refined density, and the red and green mesh show the difference density map, Fo-Fc. Bonds are depicted as lines; green for carbon atoms, red for oxygen and blue for nitrogen. Thr356 and Ser357, the activation loop residues equivalent to those targeted by RIPK3 in human MLKL, and the N- and C-lobes, are labelled. The 2Fo-Fc density was contoured at 1.0 σ and Fo-Fc density contoured at 3.5 σ. Maps were visualised in Coot[2].

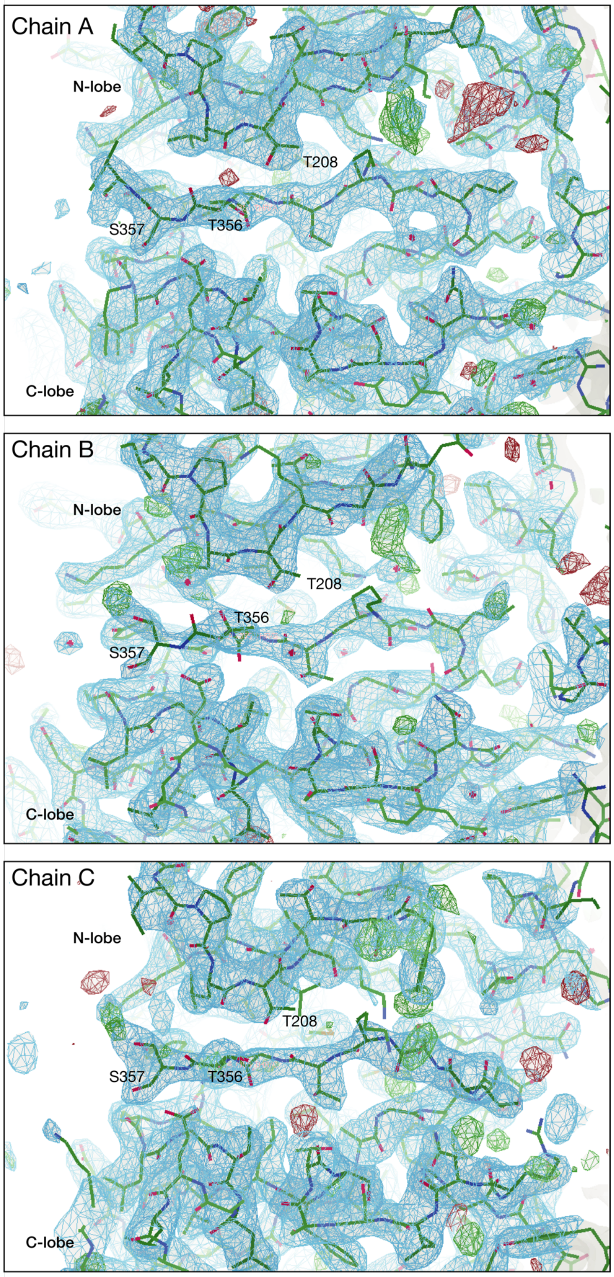

Supplementary References

1. Murphy JM*, et al.* The pseudokinase MLKL mediates necroptosis via a molecular switch mechanism. *Immunity* **39**, 443-453 (2013).

2. Emsley P, Lohkamp B, Scott WG, Cowtan K. Features and development of Coot. *Acta Crystallogr D Biol Crystallogr* **66**, 486-501 (2010).
