# Supplementary material for: Distinct pseudokinase domain conformations underlie divergent activation mechanisms among vertebrate MLKL orthologues
Source: Nat Commun. 2020 Jun 19;11:3060. doi: 10.1038/s41467-020-16823-3 (PMC7305131; doi:10.1038/s41467-020-16823-3)
Supplement: Supplementary file 3 — Reporting Summary [file 41467_2020_16823_MOESM3_ESM.pdf]

Corresponding author(s): Czabotar, Murphy

Last updated by author(s): May 27, 2020

Reporting Summary

Nature Research wishes to improve the reproducibility of the work that we publish. This form provides structure for consistency and transparency in reporting. For further information on Nature Research policies, see our Editorial Policies and the Editorial Policy Checklist.

Statistics

For all statistical analyses, confirm that the following items are present in the figure legend, table legend, main text, or Methods section.

n/a	Confirmed	
[ ]	[x]	The exact sample size (*n*) for each experimental group/condition, given as a discrete number and unit of measurement
[ ]	[x]	A statement on whether measurements were taken from distinct samples or whether the same sample was measured repeatedly
[ ]	[x]	The statistical test(s) used AND whether they are one- or two-sided<br>*Only common tests should be described solely by name; describe more complex techniques in the Methods section.*
[x]	[ ]	A description of all covariates tested
[x]	[ ]	A description of any assumptions or corrections, such as tests of normality and adjustment for multiple comparisons
[ ]	[x]	A full description of the statistical parameters including central tendency (e.g. means) or other basic estimates (e.g. regression coefficient) AND variation (e.g. standard deviation) or associated estimates of uncertainty (e.g. confidence intervals)
[ ]	[x]	For null hypothesis testing, the test statistic (e.g. *F*, *t*, *r*) with confidence intervals, effect sizes, degrees of freedom and *P* value noted<br>*Give P values as exact values whenever suitable.*
[x]	[ ]	For Bayesian analysis, information on the choice of priors and Markov chain Monte Carlo settings
[x]	[ ]	For hierarchical and complex designs, identification of the appropriate level for tests and full reporting of outcomes
[x]	[ ]	Estimates of effect sizes (e.g. Cohen's *d*, Pearson's *r*), indicating how they were calculated

Our web collection on statistics for biologists contains articles on many of the points above.

Software and code

Policy information about availability of computer code

Data collection	No software was used.
Data analysis	XDS (Version Nov 1, 2016; BUILT =20161205)was used for crystallography data processing. In Phenix 1.16, phaser-MR was used for molecular replacement and phenix.refine was used for refinement. Coot version 1.0 was used for model building.<br>FlowJo 10.1r7 was used to gate FACS data.

For manuscripts utilizing custom algorithms or software that are central to the research but not yet described in published literature, software must be made available to editors and reviewers. We strongly encourage code deposition in a community repository (e.g. GitHub). See the Nature Research guidelines for submitting code & software for further information.

Data

Policy information about availability of data

All manuscripts must include a data availability statement. This statement should provide the following information, where applicable:

- Accession codes, unique identifiers, or web links for publicly available datasets
- A list of figures that have associated raw data
- A description of any restrictions on data availability

Atomic coordinates for the rat and horse MLKL pseudokinase domains have been deposited in the Protein Data Bank with the accession numbers, 6VBZ and 6VC0 respectively (Figure 2). The data used to generate the cell death plots in Figure 1, 3 and 4 has been made available in the source data file. The uncropped blots for Supplementary Figure 3 are also included in the source data file.

Field-specific reporting

Please select the one below that is the best fit for your research. If you are not sure, read the appropriate sections before making your selection.

- [x] Life sciences
- [ ] Behavioural & social sciences
- [ ] Ecological, evolutionary & environmental sciences

For a reference copy of the document with all sections, see nature.com/documents/nr-reporting-summary-flat.pdf

Life sciences study design

All studies must disclose on these points even when the disclosure is negative.

Sample size	All experiments were performed typically with triplicate mouse cell lines or singlicate human cell lines, as noted in methods and legends, The numbers of independent repeat experiments are stated in figure legends
Data exclusions	Some death assay repeats were excluded from analysis where there was suspected technical error i.e. high levels of cell death in uninduced untreated cell lines. These datasets were excluded in entirety from analysis in these circumstances.
Replication	All experiments were repeated independently in at least triplicate, as stated in legends. In cases where there was variability between repeats more repeats were generated. With the exception of obvious technical error as stated above, all repeats were used in analysis. The natural spread of the data is shown in the paper through use of individual data points and standard error of the mean error bars.
Randomization	This is not relevant to our study. Our study compared the death of cell lines expressing different exogenes upon stimulation.
Blinding	This is not relevant to our study. Our study compared the death of cell lines expressing different exogenes upon stimulation.

Reporting for specific materials, systems and methods

We require information from authors about some types of materials, experimental systems and methods used in many studies. Here, indicate whether each material, system or method listed is relevant to your study. If you are not sure if a list item applies to your research, read the appropriate section before selecting a response.

Materials & experimental systems

n/a	Involved in the study	
[ ]	[x]	Antibodies
[ ]	[x]	Eukaryotic cell lines
[x]	[ ]	Palaeontology and archaeology
[x]	[ ]	Animals and other organisms
[x]	[ ]	Human research participants
[x]	[ ]	Clinical data
[x]	[ ]	Dual use research of concern

Methods

n/a	Involved in the study	
[x]	[ ]	ChIP-seq
[ ]	[x]	Flow cytometry
[x]	[ ]	MRI-based neuroimaging

Antibodies

Antibodies used	rat anti-MLKL (clone 3H1, produced in-house; 1:1000 dilution; available as MABC604, EMD Millipore, Billerica, MA, USA) for all MLKL blots except for pig MLKL, which was detected with rat anti-MLKL clone 5C4 (produced in-house, 1:25 dilution of unpurified hybridoma supernatant); mouse anti-Actin (A-1987, Sigma-Aldrich, St Louis, MO, USA; 1:3000); rabbit anti-GAPDH (cat#2118, Cell Signaling Technology, Danvers, MA; 1:3000); and rat anti-FLAG (clone 9H1, produced in-house; 1:1000). Secondary goat anti-mouse (cat#1010-05), goat anti-rabbit (cat#4030-05) and goat anti-rat Ig-HRP conjugates (cat#3010-05) were supplied by Southern Biotech and used at 1:5000 dilution
Validation	We previously validated anti-MLKL clone 3H1 for detection of mouse and human MLKL (Murphy et al. Immunity 2013).<br>We previously validated that anti-MLKL clone 3H1 detects horse MLKL (Tanzer et al. Cell Death Differ 2016).<br>We validate that anti-MLKL clone 3H1 can detect rat MLKL in this paper via induced vs not induced expression of an exogene encoding rat MLKL in the supplement.<br>Pig MLKL was detected with a new antibody (5C4) generated in house. We show induced vs not induced expression of an exogene encoding pig MLKL in the supplement, which validates specificity.<br>The remaining orthologs were detected by anti-flag western blots. We validated specificity of the anti-FLAG antibody by western blot on induced and uninduced flag tagged exogenes in the supplement.<br>Further details about validation of the following commercial primary antibodies:<br>* anti-GAPDH (Cell Signaling Technology, cat#2118,) - validated by manufacturer for use in ELISA, IP, IF, IHC & WB<br>* anti-Actin (Sigma #A1987) - validated by manufacturer for use in IHC, IF & WB,

Eukaryotic cell lines

Policy information about cell lines

Cell line source(s)	MLKL-deficient mouse dermal fibroblasts were sourced from MLKLko mice as described (Hildebrand et al. PNAS 2014). MLKL-deficient U937 cells are as described in Petrie et al. Nat Comm 2018 and are derived from parental cells supplied by ATCC. HEK293T cells used for lentivirus generation were sourced from ATCC. Sf21 cells were used for insect cell expression (purchased from Thermo).
Authentication	None of the cells were formally authenticated. However, their morphologies are consistent with the stated cell type. The lack of responsiveness of MLKL-deficient mouse fibroblasts and human U937 cells to necroptotic stimuli and the lack of MLKL reactivity by western blot are consistent with the absence of MLKL. Sf21 cells were susceptible to baculovirus infection and produced recombinant proteins encoded by these baculoviruses, consistent with their authenticity.
Mycoplasma contamination	All mammalian lines used were monitored for mycoplasma and were found to be negative in our routine screening.
Commonly misidentified lines (See ICLAC register)	Nil

Flow Cytometry

Plots

Confirm that:

- [x] The axis labels state the marker and fluorochrome used (e.g. CD4-FITC).
- [x] The axis scales are clearly visible. Include numbers along axes only for bottom left plot of group (a 'group' is an analysis of identical markers).
- [x] All plots are contour plots with outliers or pseudocolor plots.
- [x] A numerical value for number of cells or percentage (with statistics) is provided.

Methodology

Sample preparation	Cells were stained with propidium iodide as a marker of death. This is a very simple, routine and robust assay, which does not warrant presentation of all primary plots. Flow cytometry is used to enumerate the number of dead (PI+) cells, as illustrated in the example gating strategy presented in Supplementary Figure 2.
Instrument	FACSCalibur
Software	FlowJo 10.1r7
Cell population abundance	All data from 3 independent experiments are shown as summary statistics (number of dead cells =PI+ cells) in plots
Gating strategy	We have previously reported the gating strategy (Murphy et al. Immunity 2013). We have provided an example gating strategy as Supplementary Figure 2. Briefly cells are first gated to include only intact cells, based on forward and side scattering, then PI positive cells are gated as the 'dead' population.

- [x] Tick this box to confirm that a figure exemplifying the gating strategy is provided in the Supplementary Information.
