# Supplementary figures and images for: Distinct pseudokinase domain conformations underlie divergent activation mechanisms among vertebrate MLKL orthologues
Source: Nat Commun. 2020 Jun 19;11:3060. doi: 10.1038/s41467-020-16823-3 (PMC7305131; doi:10.1038/s41467-020-16823-3)

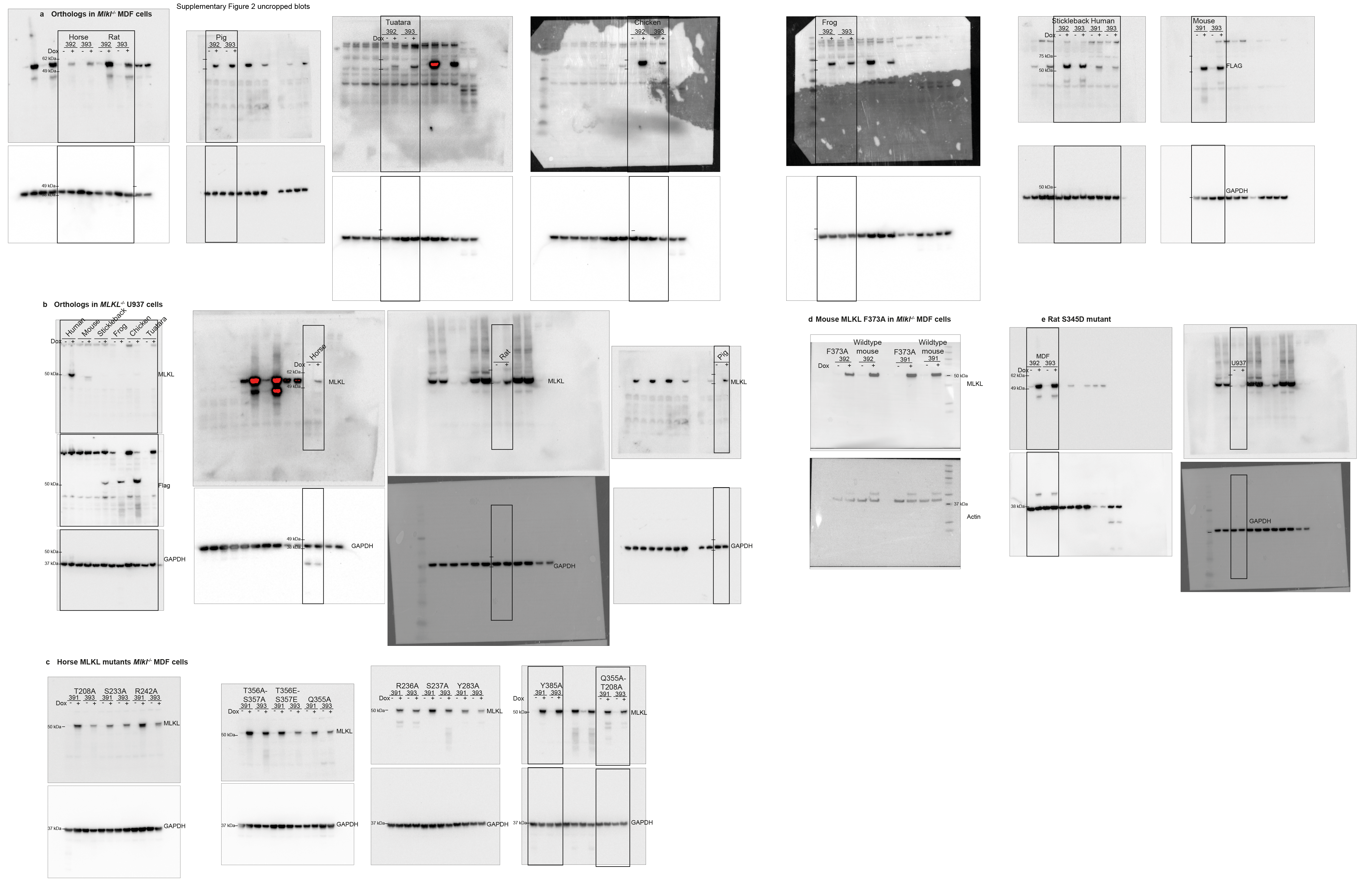

Supplement: Supplementary file 4 — Source Data [file 41467_2020_16823_MOESM4_ESM.zip › Source Data file/Revisions fully uncropped blots-01.png]
