# Supplementary material for: Distinct pseudokinase domain conformations underlie divergent activation mechanisms among vertebrate MLKL orthologues
Source: Nat Commun. 2020 Jun 19;11:3060. doi: 10.1038/s41467-020-16823-3 (PMC7305131; doi:10.1038/s41467-020-16823-3)
Supplement: Supplementary file 2 — Peer Review [file 41467_2020_16823_MOESM2_ESM.pdf]

Reviewers' comments:

Reviewer #1 (Remarks to the Author):

In this paper Davies and colleagues examine the divergence of the mechanisms for activation of the MLKL pseudokinase by its upstream regulator, the catalytically active kinase RIPK2, in different species. This signaling axis, important for initiation of the necroptosis pathway, has been demonstrated to be regulated in a species-specific manner, with no redundancy between mouse and human orthologs. By examining new crystal structures of horse and rat MLKL pseudokinases, and the ability of MLKL variants from different species to functionally complement mouse or human necroptic pathways, the authors generate new structural models for how the specificity is established.

Overall this is a very interesting and elegant work which explores a fascinating richness of the mechanisms that the MLKL-RIPK3 "cassette" has evolved in different organisms. The new structures of MLKL help to understand some of the unique features of these mechanisms, and the complementation signaling studies reveal surprising relationships between organisms from what we might have had expected. It will be gratifying one day to understand how RIPK3 kinases have evolved in different organisms to keep up with structural divergence of the MLKL modules. In sum, I am enthusiastic about this paper, and my comments below mainly concern the way the manuscript is written and how the concepts and data are introduced and explained. Careful revisions of these issues would make this manuscript more accessible to a wider audience.

Specific Points:
For the data presented in Figure 1, it is not shown what are the relative levels of MLKL variants from different species. Supplementary Figure S2 also does not provide these data, but only shows the evidence for their inducible expression. It is important to know the relative expression levels as they might contribute to the different potency of MLKL variants to induce cell death responses reported in Figure 1.

I am confused why the authors conclude that overall lack of effect of the mutations in the beta3-alphaC loop of the horse MLKL on necroptotic signaling provides the evidence for the important role of this loop in RIPK3 recognition. Isn't the opposite conclusion rather true? Wouldn't one expect that mutation of this loop should disable the ability of horse MLKL to complement mouse MLKL?

It is interesting that pig MLKL is the only other variant in the alignment shown in Supplementary Figure S1 that seems to also have the beta3-alphaC loop. It would be then predicted that pig MLKL is able to act in mouse necroptotic pathway (which to low extent seems to be the case in Figure 1) but not in human. However, pig MLKL is the only non-human MLKL tested by authors that can induce cell death in U937 cells. How do the authors explain that?

While the mutational analysis of Y384 residue in horse MLKL is elegant and demonstrates its importance for RIPK3 activation, conservation of the role of a hydrophobic residue in this position also in human and mouse MLKLs is not really an argument for species-selectivity (as it is described). Rather opposite - this seems like a general mechanism across all species. Only in the Discussion section the authors comment on other organisms, which are divergent in this region, but this is not clear as one reads the Results, and hence is confusing.

Figure 4 panels a, b, c, d and e are not referred to in the manuscript. The Results section describing this figure starts with the discussion of mutational analysis of rat MLKL activation loop, which is not shown until Figure 4e, so this seems a bit out of order. In this panel, wild type rat MLKL should be shown for reference by the way. Also, in figure legend for panel Figure 4e it is not clear that the data refers to rat MLKL. It is important to note that because so many species are discussed at the same time.

The description of the results presented in Figure 4 panels d, f and g could be made more digestible and split in at least two more paragraphs. In particular analysis of MD simulations is a little bit without context for those who are not experts in dynamics of the MLKL, and it is largely confusing. How was the entire activation loop modeled? What is the context for the conclusion that, quote, "among some MLKL orthologs, activation loop phosphorylation induces increased mobility..."?

Few typos that I caught:

Last paragraph of the Results section, it should be:
"... suggesting that addition of phosphate groups destabilizes"

First paragraph of Discussion:
It is HER3/ERBB3 not HER3/ERB3

Reviewer #2 (Remarks to the Author):

Necroptosis signalling greatly differs between difference species, but the underlying molecular reason is poorly understood. Here, Davies et al., presents some interesting data regarding the restricted ability of mouse and human RIPK3 to activate MLKL orthologs from nine vertebrates. In particular, only horse and, to some extent, pig MLKL could kill when expressed in mouse cells, whereas only pig MLKL could restore necroptotic signalling in human cells. By solving the crystal structure of horse and rat MLKL pseudokinase domain, and conducting a structure-function approach via a mutational analysis, the authors shed light into the underlaying structural basis for the observed selectivity. For instance, communication between horse MLKL and mouse RIPK3 takes place thanks to the presence of an additional short helix in horse MLKL, which occupies the position of mouse activation loop helix. The findings presented here are important because species specific differences become relevant when attempting to modulated necroptosis-mediated phathologies.

In general, this study is well controlled and conducted, and demonstrates the remarkable selectivity of RIPK3 orthologs for their cognate MLKL effectors. It also indicates that RIPK3-mediated activation of MLKL does not follow a universal molecular mechanism. Altogether, the results are novel and important as they provide strong evidence that MLKL's pseudokinase domain and RIPK3 have co-evolved between species, and are subject to divergent regulatory mechanisms.

I have only one suggestion, however this might be beyond the scope of this ms as it requires an additional structure: Given that pig MLKL can reconstitute necroptosis in human cells, it would be nice to further investigate its structure and compare it to human MLKL to elucidate the key structural features that enable this.

A few minor points should be addressed:

1 Figure 1e. Pig MLKL expressed in MLKL KO MDFs does not seem to kill very efficiently. Is this due to slower kinetics? Is higher level of cell death achieved at later time-points?

2 Figure 3 suggests that residue S232 in horse MLKL is responsible for mediating mouse RIPK3 interaction. Can the authors detect the interaction between horse MLKL and mouse RIPK3 via immunoprecipitation? If so, is this interaction compromised in S232A horse MLKL mutant cells?

3 Can the authors comment or hypothesise on why R241A horse mutant is constitutively active?

4 The authors show that R241A horse MLKL mutant is constitutively active. Does abrogation of the hydrogen bond in horse MLKL (K227-E247) also result in a constitutively active mutant, as it is the case for mouse MLKL?

5 Figure 4e. The authors do suggest that mimicking RIPK3 phosphorylation in rat is sufficient to activate rat MLKL, but the killing levels that are achieved are really low. Would this also be the case if they were to express rat phosphomimic MLKL in an Mlkl-depleted rat cell line (Eg. in rat dermal fibroblast)? Could it be that the reason why a phosphomimic mutant rat MLKL does not kill as efficiently as mouse phosphomimic MLKL is due to other factors in mouse and rat cells?

6 With regards to the increased mobility of the activation loop in horse MLKL following phosphorylation. How does this translate to the human setting? Is an increased dynamic also observed in the activation loop of human MLKL following phosphorylation?

7 Figure 2. Please rearrange the structures within Figure 2 so that they match the description of the figure legend. For instance, Figure 2a shows the structure of rat MLKL, whereas in the Figure legend 2a refers to human MLKL.

8 Within the text, in reference to Figure 4. 'Cell death was significantly elevated 2-fold… (Figure 4e). 'e' is missing.

9 Figure 4b. It seems that a residue in this figure has been mislabelled. This is C281, which according to Figure 4g and the text, should be C283.

We thank the reviewers for their positive and constructive comments, which we have incorporated into our revised manuscript. These suggestions have greatly improved the clarity of our presentation. We have addressed each reviewer comment (shown *in blue italics*) in our point-by-point response below, where we respond in plain black text.

Reviewers' comments:

Reviewer #1 (Remarks to the Author):

In this paper Davies and colleagues examine the divergence of the mechanisms for activation of the MLKL pseudokinase by its upstream regulator, the catalytically active kinase RIPK2, in different species. This signaling axis, important for initiation of the necroptosis pathway, has been demonstrated to be regulated in a species-specific manner, with no redundancy between mouse and human orthologs. By examining new crystal structures of horse and rat MLKL pseudokinases, and the ability of MLKL variants from different species to functionally complement mouse or human necroptic pathways, the authors generate new structural models for how the specificity is established.

Overall this is a very interesting and elegant work which explores a fascinating richness of the mechanisms that the MLKL-RIPK3 "cassette" has evolved in different organisms. The new structures of MLKL help to understand some of the unique features of these mechanisms, and the complementation signaling studies reveal surprising relationships between organisms from what we might have had expected. It will be gratifying one day to understand how RIPK3 kinases have evolved in different organisms to keep up with structural divergence of the MLKL modules. In sum, I am enthusiastic about this paper, and my comments below mainly concern the way the manuscript is written and how the concepts and data are introduced and explained. Careful revisions of these issues would make this manuscript more accessible to a wider audience.

We are grateful for the reviewer's enthusiastic reception of the work, and thank them for their kind words.

Specific Points:
For the data presented in Figure 1, it is not shown what are the relative levels of MLKL variants from different species. Supplementary Figure S2 also does not provide these data, but only shows the evidence for their inducible expression. It is important to know the relative expression levels as they might contribute to the different potency of MLKL variants to induce cell death responses reported in Figure 1.

We agree this is an important consideration, but regrettably experimentally not feasible to address with current reagents. There are two major confounding issues: 1. we cannot detect all the MLKL orthologs using the same antibody owing to sequence divergence (evident from alignment in Supp. Fig. 1 and Table 1); 2. where we can use the same antibody, there are differences in primary sequence that would impact affinity of the antibody for epitope sequence. Consequently, we have been careful to report the expression of the MLKL orthologs as detectable or not, rather than inferring relative quantities of protein expression. We have now made comment in the main text to address the reviewer's important point, on line 119-124.

I am confused why the authors conclude that overall lack of effect of the mutations in the beta3-alphaC loop of the horse MLKL on necroptotic signaling provides the evidence for the important role of this loop in RIPK3 recognition. Isn't the opposite conclusion rather true? Wouldn't one expect that mutation of this loop should disable the ability of horse MLKL to complement mouse MLKL?

We apologize for not making our reasoning clear. We have included further text on page 7 (lines 176-182, 188-190) to clarify our rationale. In essence, the key residue that superimposes with mouse MLKL S228, which is a RIPK3 contact residue in the Xie *et al*. mouse MLKL:RIPK3 structure, is horse MLKL S233 within the β3-αC loop. Because mutation of horse MLKL S233 to Ala compromised reconstitution of signaling in mouse cells, this argues for a comparable role for horse MLKL S233 and mouse MLKL S228 enabled by conservation of their spatial position.

It is interesting that pig MLKL is the only other variant in the alignment shown in Supplementary Figure S1 that seems to also have the beta3-alphaC loop. It would be then predicted that pig MLKL is able to act in mouse necroptotic pathway (which to low extent seems to be the case in Figure 1) but not in human. However, pig MLKL is the only non-human MLKL tested by authors that can induce cell death in U937 cells. How do the authors explain that?

This is an interesting point, although without the pig MLKL structure, it is something we feel we are unable to definitively address here. Based on primary sequence, we agree it is possible that the pig MLKL β3-αC loop (sequence SQASD) might form a helix like in horse MLKL (SQARS). However, this helix is especially dynamic in our MD simulations, which leads us to speculate that the structural context for the helix is an important factor in its stabilization. Additionally, it is not clear whether the Asp vs Arg charge in pig vs horse sequence might impact helicity or stabilizing interactions. These factors limit our capacity to predict whether this helix might occur in other MLKL orthologs, and illustrate the value of further structural studies. Regardless, it is likely this site contributes to pig MLKL's capacity to be activated by human RIPK3 in U937 cells, in addition to the activation loop and the C-lobe aromatic residue. We have elaborated on the potential helicity of the pig MLKL β3-αC loop and its possible contribution to human RIPK3 binding, noting the appropriate caveats, on page 11 of the revised manuscript. (lines 301-307).

While the mutational analysis of Y384 residue in horse MLKL is elegant and demonstrates its importance for RIPK3 activation, conservation of the role of a hydrophobic residue in this position also in human and mouse MLKLs is not really an argument for species-selectivity (as it is described). Rather opposite - this seems like a general mechanism across all species. Only in the Discussion section the authors comment on other organisms, which are divergent in this region, but this is not clear as one reads the Results, and hence is confusing.

We apologize for confusing this matter. We entirely agree with the reviewer that the conserved C-lobe Tyr/Phe is unlikely to be a determinant of species selectivity, although it is clearly involved in RIPK3 binding. We have taken the reviewer's comments on board and have revised text in the Results accordingly on page 8, lines 202-205 and in Discussion (page 11, lines 315-317).

Figure 4 panels a, b, c, d and e are not referred to in the manuscript. The Results section describing this figure starts with the discussion of mutational analysis of rat MLKL activation loop, which is not shown until Figure 4e, so this seems a bit out of order. In this panel, wild type rat MLKL should be shown for reference by the way. Also, in figure legend for panel Figure 4e it is not clear that the data refers to rat MLKL. It is important to note that because so many species are discussed at the same time.

The description of the results presented in Figure 4 panels d, f and g could be made more digestible and split in at least two more paragraphs.

We thank the reviewer for this suggestion. We agree the order of panels in Figure 4 could be more logical and have reordered in light of the reviewer's comments. In line with the reviewer's suggestion, we have taken the opportunity to add more text to describe the panels of Figure 4 (lines 221-228; 258-265) and to add more detail to the Figure 4a legend to ensure complete clarity.

In particular analysis of MD simulations is a little bit without context for those who are not experts in dynamics of the MLKL, and it is largely confusing. How was the entire activation loop modeled?

We apologize for our brevity in this description. We have now added additional detail to provide greater context for the MD simulations and more detail of how the activation loop was modelled. We have expanded our description of the MD findings (page 9, lines 254-256 and 258-265) and now describe how the missing residues were modelled in the Results (lines 250-251).

What is the context for the conclusion that, quote, "among some MLKL orthologs, activation loop phosphorylation induces increased mobility..."?

Coupled with the suggestions of reviewer 2, we have taken the opportunity to reword to ensure our reasoning is clear and that it is clear this is an hypothesis (page 9, paragraph before Discussion).

Few typos that I caught:

Last paragraph of the Results section, it should be:
"... suggesting that addition of phosphate groups destabilizes"

We thank the reviewer for their careful reading of the manuscript. We have revised accordingly.

First paragraph of Discussion:
It is HER3/ERBB3 not HER3/ERB3

Corrected, with thanks.

Reviewer #2 (Remarks to the Author):

Necroptosis signalling greatly differs between difference species, but the underlying molecular reason is poorly understood. Here, Davies et al., presents some interesting data regarding the restricted ability of mouse and human RIPK3 to activate MLKL orthologs from nine vertebrates. In particular, only horse and, to some extent, pig MLKL could kill when expressed in mouse cells, whereas only pig MLKL could restore necroptotic signalling in human cells. By solving the crystal structure of horse and rat MLKL pseudokinase domain, and conducting a structure-function approach via a mutational analysis, the authors shed light into the underlaying structural basis for the observed selectivity. For instance, communication between horse MLKL and mouse RIPK3 takes place thanks to the presence of an additional short helix in horse MLKL, which occupies the position of mouse activation loop helix. The findings presented here are important because species specific differences become relevant when attempting to modulated necroptosis-mediated phathologies.

In general, this study is well controlled and conducted, and demonstrates the remarkable selectivity of RIPK3 orthologs for their cognate MLKL effectors. It also indicates that RIPK3-mediated activation of MLKL does not follow a universal molecular mechanism. Altogether, the results are novel and important as they provide strong evidence that MLKL's pseudokinase domain and RIPK3 have co-evolved between species, and are subject to divergent regulatory mechanisms.

We greatly appreciate the reviewer's positivity towards our work.

I have only one suggestion, however this might be beyond the scope of this ms as it requires an additional structure: Given that pig MLKL can reconstitute necroptosis in human cells, it would be nice to further investigate its structure and compare it to human MLKL to elucidate the key structural features that enable this.

Since submission of our manuscript, we have committed ourselves to expressing and purifying the pig MLKL pseudokinase domain from insect cells with the goal of crystallizing the protein, in line with the reviewer's suggestion. This has required a number of iterations: the C-terminus of pig MLKL contains two non-conserved Cys residues, which we have now eliminated to improve expression of soluble protein; codon optimization was also required to express satisfactory amounts of soluble protein from insect cells. While we have now got to the point where we can express reasonable yields of the pig MLKL pseudokinase domain from Sf21 cells, the protein has proven extremely unstable. We see a lot of precipitation during purification and, upon concentrating gel filtration fractions, the protein crashes. So far, we have only achieved a maximum of 0.4mg/mL concentration. We have performed a buffer optimization screen for thermal stability; the protein has a respectable Tm of ~44°C, but this was not enhanced by changing pH, salt concentration or additives. Whether changing these conditions might minimize aggregation/precipitation awaits further (extensive) trial-and-error experimentation, which we consider likely to be a longer term endeavour. We

expect we need to optimize protein behaviour to allow concentration to 4-5mg/mL (and monodispersity) for crystal trials, which will be our focus at the conclusion of the COVID-19 shutdown.

A few minor points should be addressed:

1 Figure 1e. Pig MLKL expressed in MLKL KO MDFs does not seem to kill very efficiently. Is this due to slower kinetics? Is higher level of cell death achieved at later time-points?

The goal of these experiments was to deduce whether mouse RIPK3 can "talk" to MLKL from other species. For this reason, we took the 24 hour timepoint to compare with mouse MLKL reconstitution of death in *Mlkl*$^{-/-}$ MDFs, as at this timepoint exogenous mouse MLKL enables 80% death following application of a necroptotic stimulus. It is entirely possible that by extending the timepoint to 48 hours, we would see additional necroptosis mediated by pig MLKL, although our thinking is that it is the imperfect compatibility of mouse RIPK3 and pig MLKL that underlies the deficits in death. Regrettably, owing to the COVID-19 crisis, we cannot formally examine the kinetics of cell death mediated by mouse vs pig MLKL in *Mlkl*$^{-/-}$ MDFs at this time. In light of the reviewer's query, we have included additional description of the possibilities raised by the reviewer in our revised Results section (page 5, lines 124-127).

2 Figure 3 suggests that residue S232 in horse MLKL is responsible for mediating mouse RIPK3 interaction. Can the authors detect the interaction between horse MLKL and mouse RIPK3 via immunoprecipitation? If so, is this interaction compromised in S232A horse MLKL mutant cells?

This is a very good point, albeit one not readily addressable using the reagents we have to hand. Our experience indicates that endogenous mouse MLKL does not IP mouse RIPK3 from MDF cells (Murphy *et al*., *Immunity* 2013), which led us to propose that there is a transient interaction between mouse RIPK3 and MLKL that activates MLKL via a "kiss-and-run" mechanism (e.g. in Petrie *et al*., *TIBS* 2019). Even in the case of human cells, where our earlier data support a stable interaction of RIPK3 and MLKL (e.g. Petrie *et al*., *Nat Comm* 2018), our available antibodies are not suitable for MLKL IP. It was only our recent development of human MLKL-specific Monobodies (Petrie *et al*., *PNAS* 2020) that has enabled us to successfully and selectively IP human MLKL in the past few months. Examining the horse MLKL interaction with mouse RIPK3 is even more complicated in the present study, because we do not have any suitable validated antibodies to enable IP of horse MLKL and associated RIPK3. Availability of such antibodies in future will allow for these experiments, but regrettably they cannot be performed for this manuscript.

3 Can the authors comment or hypothesise on why R241A horse mutant is constitutively active?

We thank the reviewer for prompting further discussion on this. We have now added further text to describe R242's interactions (Results, lines 178-182) and speculate on the possible mechanisms underlying the constitutive killing activity in the Discussion (lines 337-348).

4 The authors show that R241A horse MLKL mutant is constitutively active. Does abrogation of the hydrogen bond in horse MLKL (K227-E247) also result in a constitutively active mutant, as it is the case for mouse MLKL?

This is a good question. In mouse MLKL, the situation is quite different to human and horse MLKL. In mouse MLKL structures, the interaction is between the β3 (VAIK motif) Lys, K219, and the unconventional activation loop helix residue Q343, rather than a more conventional αC Glu, E239. Disruption by mutation or K219, Q343 or adjacent S345 is sufficient to trigger mouse MLKL killing. In the human and horse MLKL structures, as the reviewer notes, there is a conventional VAIK Lys:αC Glu interaction. As such, we would not anticipate constitutive activity, because disruption of the human MLKL K230:E250 interaction is insufficient to trigger constitutive killing. We have added additional details in the Results section where we compare the differences between the ortholog structures to ensure these differences are clearly described (on lines 148-152). We have added additional text (lines 221-227) to underscore that horse MLKL is more

human MLKL-like, than similar to the mouse structure.

5 Figure 4e. The authors do suggest that mimicking RIPK3 phosphorylation in rat is sufficient to activate rat MLKL, but the killing levels that are achieved are really low. Would this also be the case if they were to express rat phosphomimic MLKL in an Mlkl-depleted rat cell line (Eg. in rat dermal fibroblast)? Could it be that the reason why a phosphomimic mutant rat MLKL does not kill as efficiently as mouse phosphomimic MLKL is due to other factors in mouse and rat cells?

This is a very good point. We have now added further text to convey this possibility in Discussion (lines 364-366, pages 12-13). It is worth noting that the identities of essential factors, and thus the bounds of species specificity, beyond the RIPK1:RIPK3:MLKL components is yet to be established. As a result, we cannot state with certainty whether there are additional factors that are peculiar to rat cells that would facilitate greater constitutive killing of by rat MLKL phosphomimetic mutants in rat cells relative to mouse cells. One possibility is that recruitment to rat RIPK3 may contribute to the assembly of higher order signaling complexes, although such an idea awaits formal examination in future work.

6 With regards to the increased mobility of the activation loop in horse MLKL following phosphorylation. How does this translate to the human setting? Is an increased dynamic also observed in the activation loop of human MLKL following phosphorylation?

This is an excellent question, which was also touched on by Reviewer 1. Here, we address this query for the benefit of the reviewers, although we have elected to omit from the revised text owing to the number of caveats associated with such a simulation (unless the reviewers and editor feel strongly about its inclusion). Principally, we are concerned that to perform MD simulations of the effect of phosphorylation on the human MLKL activation loop, we needed to model the region in which the phosphosites reside and then examine the effect of phosphorylation on mobility. While we see increased mobility in a 280ns simulation of the dephospho- vs pT357/pS358 human MLKL structure (Response Figure 1), consistent with the idea of increased mobility on the activation loop upon phosphorylation, this requires substantial speculation in the absence of a human MLKL structure with an ordered activation loop. We plan to examine this more closely in future when we have human MLKL experimental structures containing an ordered activation loop to hand, such as might be achievable by co-crystallizing human MLKL pseudokinase domain with monobodies, Fabs or human RIPK3. However, for now, we would prefer to isolate our discussion of MD simulations to the horse MLKL structure reported for the first time, in which the residues subject to phosphorylation by RIPK3 are ordered.

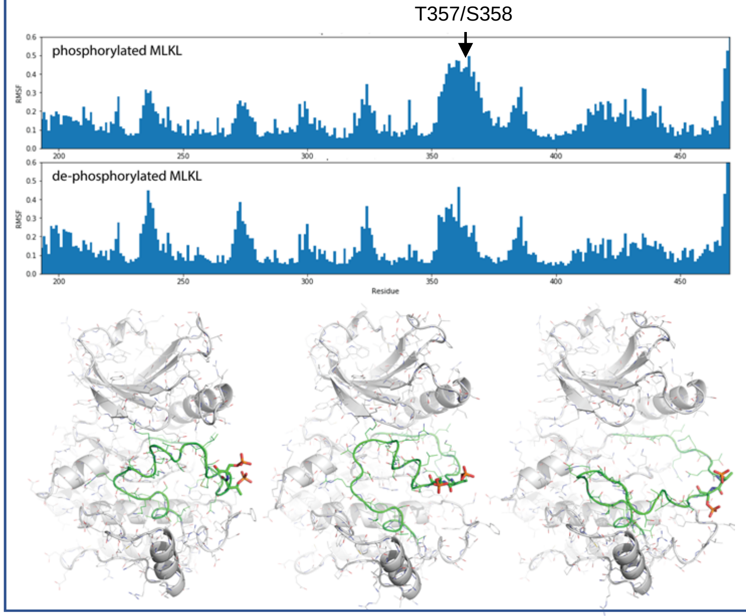

Response Figure 1. 280 ns molecular dynamics simulations of human MLKL pseudokinase domain (PDB 4MWI) with modelled activation loop.

Upper panels: simulations of pT357/pS358 vs dephospho- human MLKL shows increased activation loop mobility on phosphorylation.

Lower panels: snapshots of the trajectory of activation loop thermal fluctuations during MD simulations of the phospho- human MLKL pseudokinase domain. The activation loop is shown in green and pT357 and pS358 as sticks. It is notable that there is no charged interaction that anchors the phospho-activation loop to the core domain. The significance of this will be examined in future when human MLKL structures containing ordered activation loop are available.

7 Figure 2. Please rearrange the structures within Figure 2 so that they match the description of the figure legend. For instance, Figure 2a shows the structure of rat MLKL, whereas in the Figure legend 2a refers to human MLKL.

We thank the reviewer for picking this up, and apologize for this oversight. We have now corrected the Figure 2 layout to match the originally-submitted legend.

8 Within the text, in reference to Figure 4. 'Cell death was significantly elevated 2-fold… (Figure 4e). 'e' is missing.

Thanks once more for the reviewer's careful reading of our manuscript. We have now updated to "Figure 4a" in line with the reordering of panels during revision.

9 Figure 4b. It seems that a residue in this figure has been mislabelled. This is C281, which according to Figure 4g and the text, should be C283.

Corrected, with thanks. We also wish to note that we identified a shift in amino acid numbering in the course of revision, where we had misnumbered the horse MLKL residues out of sequence by one. We have corrected the figures, text and PDB file accordingly; the numbering now corresponds to the PubMed protein database sequence numbering, which was recently updated.

REVIEWERS' COMMENTS:

Reviewer #1 (Remarks to the Author):

The authors have satisfactorily addressed all my comments.

Reviewer #2 (Remarks to the Author):

The authors have addressed my concerns, where this was possible under the current situation, and I am happy with their manuscript.
